# Climate change and citrus nutrition: mechanisms, nutrient imbalances, and inclusive management

**DOI:** 10.3389/fpls.2026.1798826

**Published:** 2026-05-20

**Authors:** Anoop Kumar Srivastava, Neriman Tuba Barlas, Qiang-Sheng Wu, Eran Raveh, Gustavo Brunetto, Tripti Vashisth, Seyed Majid Mousavi, Umesh Kumar Acharya

**Affiliations:** 1 Indian Council of Agricultural Research-Central Citrus Research Institute, Nagpur, Maharashtra, India; 2 Agricultural Faculty, Soil Science and Plant Nutrition Department, Ege University, Izmir, Türkiye; 3 College of Horticulture and Gardening, Yangtze University, Jingzhou, China; 4 Agricultural Research Organization (ARO), Rishon LeZion, Israel; 5 Department of Soil Science, Federal University of Santa Maria, Santa Maria, Brazil; 6 Citrus Research and Education Center, University of Florida, Gainesville, FL, United States; 7 Soil and Water Research Institute, Agricultural Research, Education, and Extension Organization, Karaj, Iran; 8 National Commercial Crop Research Center, Nepal Agricultural Research Council, Lalitpur, Nepal

**Keywords:** citrus, climate change, drought, elevated CO2, fertigation, fruit quality, Huanglongbing, nutrient-use-efficiency

## Abstract

Climate change as a global issue is increasingly redefining the nutritional constraints in citriculture, thereby increasing the reliance on optimized use of nutrients and irrigation, while rising atmospheric CO_2_ continues to influence the carbon(C)–nutrient balance. Citrus is an evergreen perennial with relatively shallow root system and prolonged fruit development period, the abiotic stresses interact across seasons to alter soil nutrient supply, root nutrient uptake kinetics, whole-tree mineral status, and eventually the fruit nutritional quality. We synthesized current evidences on climate-driven pathways affecting citrus nutrition, impact of high temperature, and water scarcity restricting nutrient mobility and acquisition, nutrient leaching due to heavy rainfall, poor aeration-induced anoxia-related nutrient constraints, and salinization disrupting ionic homeostasis via elevated (sodium/potassium ratio) Na/K and (sodium/calcium ratio) Na/Ca ratios in plant roots. We further collated multi-point facts, that elevated CO_2_ stimulated biomass increase, yet dilutes tissue nutrient concentration, thereby increasing risks for nutrient deficiencies in citrus production system. Across regions, in economic terms, climate change translates into yield instability, fruit grade loss, and adding extra cost investments on irrigation and nutrient management. Finally, we identified priorities for climate-resilient citrus nutrition, as: monitoring-based diagnostics, precision fertigation using 4R (right source, right time, right rate, and right place) strategies, soil organic carbon–centered integrated soil fertility management (ISFM), phytobiome manipulation for microbial consortia development, and nutriomics-mediated climate proofing of rootstock–scion combinations. Together, these approaches provide a conceptual framework to sustain citrus productivity and fruit nutritional quality under current conundrum of climate change.

## Introduction

Citrus is one of the highly researched fruit crops worldwide. A global meta-analysis of yields, water use-efficiency (WUE), and nitrogen-use-efficiency of citrus production systems, using 1009 observations from 55 studies, covering 11 countries showed median citrus yields ranging from 30 to 60 Mg ha^-1^, falling within the average global yields of 10–30 Mg ha^-1^ and attainable yields of 60–90 Mg ha^-1^, coupled with median WUE ranged from 2.5 to 5 kg m^-3^ and median nitrogen-use-efficiency from 150 to 350 kg kg^-1^ (Qin et al., 2016). Of late, both WUE and nutrient-use-efficiency (NUE) have been severely challenged by global phenomenon of climate change.

Climate change refers to dynamic shifts in temperature regimes, precipitation patterns, frequency and intensity of extreme events, coupled with greenhouse gas emissions. Global mean temperature has already risen by roughly 1.1–1.3 °C relative to pre-industrial period, with measurable consequences on soil-plant-environment-human health (Brasseur et al., 2025). These impacts are increasingly associated with more frequent and severe heatwaves, droughts, floods, and storms that disrupt livelihoods and generate substantial economic losses (Abbass et al., 2022). Although, mitigation and adaptation policies are expanding, current trajectories suggest the urgency of integrated responses that combine rapid emissions reductions along with locally tailored adaptations.

Sustaining quality production of fruit crops is amongst the most climate-sensitive sector, severely constrained by rising temperature, salinity, water availability extremes as drought or waterlogging, and pest–disease dynamics (e.g. HLB, Huanglongbing known as citrus greening disease). Climate change has slowed down the pace of yield gains, reducing the productivity in many tropical and subtropical regions. Projections indicate further yield losses with rise in temperature extremes, drought, salinity, and rainfall variability (Ortiz-Bobea et al., 2021). Crop yield increases under elevated atmospheric CO_2_ through stimulated photosynthesis in C_3_ species, are often offset by heat stress, accelerated phenology, rising evaporative demand, and nutrient dilution effects, thereby reducing the nutritional quality (Gojon et al., 2022; Bibi and Rahman, 2023).Within this context, citrus widely cultivated across tropical and subtropical regions, occupies a vulnerable position. Various case studies indicate that heat, drought, excess rainfall, storms, and salinity increasingly reduce photosynthesis and carbohydrate accumulation, promote fruit drop, and increase sunburn and rind disorders effects lowering marketable yield (Ahmed et al., 2025). While, high temperature may increase climatic suitability in some temperate regions, potential yield gains are frequently coupled with inferior quality and intensive management requirements (Barlas and Kadyampakeni, 2022; Wang et al., 2022).

A key reason for the high sensitivity of citrus ecosystems is the vulnerability of citrus nutrition to climate stresses. As an evergreen perennial with long-lived foliage and relatively shallow roots, citrus depends on stable soil moisture and moderate temperatures to sustain nutrient uptake and canopy carbon balance proper fruit development (Medda et al., 2022). High-temperature induced, photoinhibition-driven, oxidative stress limits nutrient assimilation and transport (Naeem et al., 2024). Drought on the other hand, reduces mass flow and diffusion of nutrients, whereas high rainfall aiding leaching of mobile nutrients such as nitrate (NO_3_) and magnesium (Mg), particularly in sandy soils, creating paradox elevated nutrient demand under reduced nutrient acquisition capacity (Kadyampakeni et al., 2018). These interacting mechanisms ultimately manifest in inferior fruit quality with low nutritional value by bringing changes in sugars, organic acids, vitamin-C, antioxidant composition, and mineral profiles driven further by altered phenology, carbohydrate allocation, water relations, and root–soil processes (Gojon et al., 2022; Mesejo et al., 2024; Auñón-Calles et al., 2025). The economic implications of climate change induced responses therefore extend far beyond yield and fruit quality to encompass marketability and production costs, where nutritional traits influence price premiums and processing value. At the same time, adaptation often requires investment in irrigation modernization, salinity control, and targeted fertilization, increasing exposure to water scarcity, energy prices, and input markets (Mushtaq et al., 2024).

Fertilizers are the major production cost component, which dominate the environmental cost profiles. Citrus nutrition management is, therefore, increasingly governed by climate-related policy and regulatory pressures. In this backdrop, a global perspective is essential, since prevalence of climate risks vary widely amongst citrus regions. Many water-limited production systems face increasing frequency of heatwaves, drought, salinity, and compound stresses that adversely affect both productivity and nutritional quality. While socio-economic capacity (credit, insurance, extension, and climate information) governs the feasibility and speed of adaptation (Karamidehkordi et al., 2023). Understanding climate impacts on citrus nutrition warrant at linking biophysical drivers to management feasibility and economic constraints across heterogeneous production contexts. This review aims to synthesize climate change affecting citrus nutrition and translate emerging insights into actionable priorities for climate-resilient management. With this background information, we: i. summarize major climate stressors shaping citrus nutrition and underlying mechanistic pathways; ii. evaluate observed and projected effects on nutrient uptake, partitioning, mineral imbalances, and key fruit nutritional traits; and iii. identify priorities for climate-smart citrus nutrition, including monitoring-based diagnostics, precision fertigation, ISFM, and rootstock–scion choices against climate change-related constraints. By addressing these objectives, this review provides a conceptual framework to guide research and management decisions for sustaining citrus nutritional quality and orchard profitability under climate change scenario.

## Identification of knowledge gaps through bibliometric analysis

In order to map research trends and identify research gaps, bibliometric analyzes reveal that only 178 climate–citrus related studies published in scopus-indexed journals between 1992 and 2022, with research primarily distributed across modeling, socio-political dimensions, and plant physiology. However, studies integrating these research domains are very limited. Our analysis identifies two critical gaps from the published literature, viz, i. crop response in relation to combined abiotic and biotic stresses (e.g., heat + drought + salinity + HLB), with severely limited field response functions (Rasera et al., 2023) and ii. lack of predictive models capable of projecting yield and quality across citrus regions and climate scenarios, largely site- or period-specific (Wang et al., 2022). Subsequent published literature extending through 2023–26 confirm a shift from abstract risk modeling towards individual stress-focused research, addressing drought, heat stress, irrigation demand, and plant physiological performance under increasingly variable climatic conditions (Hussain et al., 2023; Dong et al., 2024; Osorio-Marın et al., 2024).

**Figure 1:**
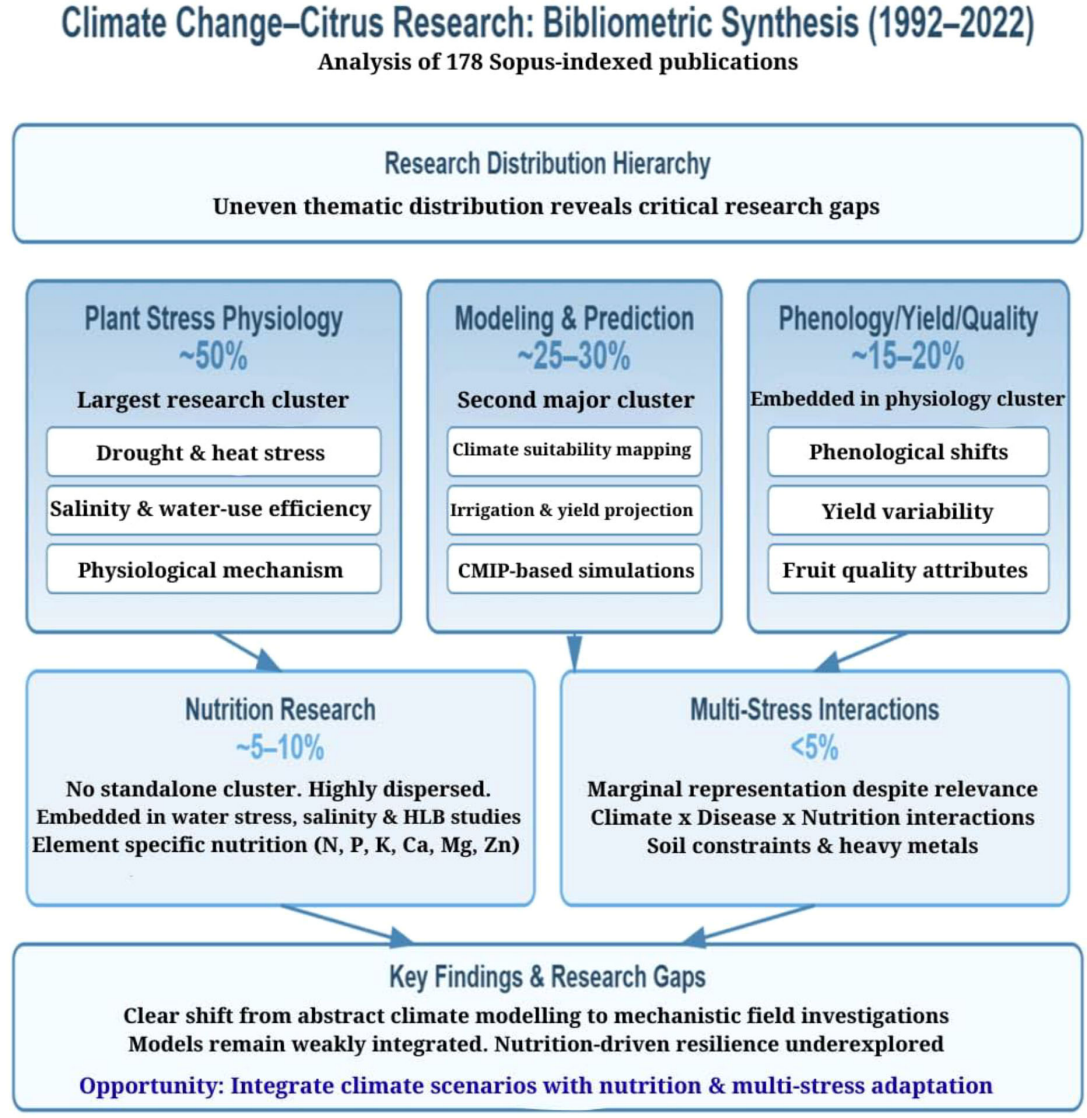
Bibliometric analysis of climate change -citrus nutrition response relationship.

Within plant physiology and stress-response (cluster-I), phenology, yield fluctuation, and fruit quality remain the most intensively studied research areas (**Figure 1**). Previous studies document climate-driven changes in phenology of flowering, fruit set, fruit size development, sugar–acid balance, peel integrity, and interannual yield variability across major citrus-growing regions (Shafaqat et al., 2021; Wang et al., 2022; Hussain et al., 2023; Bacelar et al., 2024).

These physiological events continue to serve as primary response indicators in current bibliometric mappings. Modeling and prediction studies constitute a second major issue as cluster-II, still an underdeveloped research axis. Existing works focus primarily on climate suitability, water demand, and to a lesser extent, yield and quality projections using statistical regression approaches, coupled with model intercomparison time-series forecasting methods (Khan and Chunjie, 2026). More recently, machine-learning and digital modeling frameworks have emerged, however only with limited field success as cluster-III (Wang et al., 2022; Osorio-Marın et al., 2024). Mineral element-specific nutrition remains dispersed across general plant nutrition, stress mitigation, or disease management studies, and is rarely addressed within climate change–citriculture framework. Nutritional issues are most visible where they intersect with water stress, salinity, or disease pressure, particularly HLB, rather than as standalone climate–nutrition research topic. Literature published through 2024– early 26 (Jan-Mar) identifies limited insights on various issues of balanced fertilization under changing climate, as key barriers to the evolvement of well-developed strategies about climate- nutrition based adaptation for citrus production systems. Similarly, combined abiotic–biotic stress interactions, such as climate x HLB x nutrition x heavy metal dynamics is another unexplored research area (Dong et al., 2021; Rasera et al., 2023; Bacelar et al., 2024). Hence, we previously identified two critical research gaps, which are further split into theme-based gaps as: nutrient-use-efficiency (NUE) and WUE with and without chemical fertilizers (foliar nutrition, organic fertilizers use, and ISFM supported with renewed efforts on nutrient diagnostics) in presence or absence of HLB, microbial manipulation for consortia development, rootstocks intervention, use of soil aggregators like AMF (arbuscular mycorrhizal fungi) and biochar for soil health improvements cascading into soil carbon loading, superior fruit nutritional traits, and high nutrient density, in addition to moderating adaptation barriers under simultaneous multiple climate stressors.

## Citrus performance under climate change

Citrus represents long-lived and evergreen plants. The quality parameters of high-value fruits such as sugar–acid balance, vitamins, and secondary metabolites are controlled by climate (Dong et al., 2024). Combined climate stresses are more damaging than single stress, sharply reducing photosynthesis, stomatal conductance, fruit set, fruit size, and sugar–acid balance in citrus. Multi-year field studies show that high temperatures and rainfall patterns strongly alter soluble solids (sugars) and titratable acidity in sweet oranges and mandarins, changing flavor, and internal quality (Rodríguez-Azorín et al., 2025). In arid climate, days with temperature >35 °C (10 to 50 days) was associated with lower juice and sugar content, raising the concentration of organic acids, and degrading the quality of citrus fruits (Mesejo et al., 2024).

Climatic stresses like drought, heat, salinity, and waterlogging, all modify root functions and mineral uptake. Citrus, despite displaying high sensitivity to salinity and boron (B)-deficiency, shows rootstock-dependent responses when irrigated with desalinated seawater under high temperature (37/25^0^C), with growth reductions linked to toxic ion accumulation and oxidative stress (Navarro et al., 2022; Qaderi et al., 2025). Internal fruit quality development in terms of accumulation of acids and sugars, is particularly temperature-sensitive during fruit ripening stages (Medda et al., 2022; Hussain et al., 2023). Key metabolic traits and nutritional quality of citrus fruits under climate change scenario is further summarized (**Table 1**). Hence, temperature and rainfall reshape fruits sugars, acids, and nutrients uptake along with physiological response in long annual growth cycle of citrus crop.

**Table 1:** Key citrus metabolic traits and their sensitivity to climate change-driven nutritional parameters.

Vulnerable features	Response attributes	References
Long fruiting period and perennial canopy	Multi-year climate stresses reduce fruit nutrients composition	Hussain et al. (2023); Mesejo et al. (2024)
Sugar–acid balance of fruits	Small increase in temperature/rainfall changes the taste and marketability	Mesejo et al. (2024); Dong et al. (2024)
Canopy sensitivity	Stress-driven changes reduce accumulation of minerals, antioxidants, and organic acids	Hussain et al. (2023); Qaderi et al. (2025)
Rootstock-dependence	Choice of rootstock alters nutrient uptake and stress metabolites	Medda et al. (2022)

Nine-year field response studies (2014-22) showed that low rainfall (<220 mm), high cumulative temperature (>3150 °C), and diurnal temperature variation (>14 °C) promoted the accumulation of soluble solids (>9%) in young fruits at 120-days after flowering, thus affecting the nutritional and organoleptic quality of Bingtang orange (*Citrus sinensis* L. Osbeck, cv. Bingtang) (Dong et al., 2024). Heat and drought also alter uptake and internal use of key nutrients like N, potassium (K), calcium (Ca), and Mg, affecting antioxidants and vitamin-related metabolism (Gupta et al., 2024; Bacelar et al., 2024). Studies further highlight the role of improved nutrient management and rootstock, choice, coupled with precision irrigation as main levers to stabilize citrus nutritional quality under climate change (Soares et al., 2019; Hussain et al., 2023). The emerging priorities and technical directions required to support resilience of citrus to ensue yield stability and fruit quality under future climates are summarized (**Table 2**), with an emphasis on climate-smart nutrition program.

**Table 2:** Key areas of citrus nutrition requiring climate resilience.

Key issues	Response function under climate change	References
Stress-based fertilization (N, K, Ca, Mg, B, and Mn)	Improves stomatal control, water status, ROS-detoxification and heat/drought tolerance	Gupta et al. (2024); Bacelar et al. (2024)
Rootstock–based nutrients accumulation	Use of rootstocks tolerant to high salt, B and drought, keeping toxic ions low with nutrient balance	Vincent et al. (2020)
Soil and water management (mulch, no-tillage, biofertilizers, AMF)	Aid in better root growth, water/nutrient uptake, and antioxidant capacity under drought	De Souza et al. (2025)
Decision tools and models linking climate–plant nutrition–fruit quality	Predict fruit quality/yield under future climates and guide balanced fertilization	Rasera et al. (2023)

N, K, Ca, Mg, B, and Mn denote nitrogen, potassium, calcium, magnesium, boron, and manganese, respectively. ROS and AMF stand for reactive oxygen species and arbuscular mycorrhizal fungi, respectively.

## Nutrient uptake and partitioning over tree lifespan

### Mineral imbalances and fruit nutritional traits

Citrus fruits are nutrient-dense powerhouses characterized by high vitamin-C (ascorbic acid), dietary fiber, and bioactive compounds like flavonoids (hesperidin, naringin) and carotenoids (Razi et al., 2011). These traits are achievable through nutritional balance, the backbone of crop performance and orchard longevity. Low N or phosphorous (P) is reported to distort nutrient balance, root/shoot ratios, and photosynthetic efficiency, with adaptive shifts maximizing prioritized allocation of nutrients to roots and improved photosynthetic NUE (Meng et al., 2021; Huang et al., 2021).

Studies by Alva et al. (2006b) showed that fruit- N correlated with fruit load, thus, at a given N-rate, N-removal by fruit was lower during years of low fruit yield as compared to years of high fruit yield. In high fruit production years, fruit-N accounted for about 45% of total N input on an annual basis. As significant as 15% of total N input at 280 kg N ha^−1^ year^−1^ was not accounted in N-budget, due to leaching loss. Under N-deficiency or disease incidence, trees adapt by modifying their physiology, biomass, and nutrient allocation toward roots and or defense-related metabolites, altering both nutrient-uptake-efficiency and fruit nutritional traits (Atta et al., 2022; Fajardo et al., 2025). Likewise, amongst micronutrients, B-auxin association showed hormonal- and membrane-level processes regulating root architecture and plasma-membrane H^+^-ATPase (an enzyme catalyzing conversion of adenosine triphosphate into adenosine diphosphate and inorganic phosphate), governing the nutrient uptake differentially expressed as per rootstocks (Ferreira et al., 2020; Yang et al., 2025). Such mechanisms and consequences of mineral imbalances are, however, not integrated into a long-term nutrient allocation study across the tree’s lifespan.

Nutritional traits of fruits are thus widely reported, including the metabolite-based quality assessment (Lado et al., 2018; Guo et al., 2023), but rarely connect to issues like nutrient uptake and partitioning on whole-tree basis. Issues connecting temporal scale distribution of nutrients from juvenile to reproductive phase, on-year versus off-year, and periods of senescence, are sparsely studied. Linking fruit-level traits to whole-tree nutrient partitioning and allocation/reallocation of nutrients pool within a life time of citrus trees, is rarely enumerated.

### Partitioning of nutrients

Citrus trees have the ability to prioritize allocation of freshly absorbed nutrients to new shoots and developing fruits during active growth and fruiting phase, with old leaves and woody organs acting as important nutrient reserves (Lu et al., 2021). Dynamics of seasonal patterns and N-reserves showed spring time application of ¹^5^N in young bearing grapefruit (*Citrus paradisi* Macfad.) indicated that new leaves and fruits were dominant sinks (storing 40–70% of spring-applied N). While older leaves (> 5-months-old) and woody tissues act as N- reserves remobilized to support new flush of leaves and fruit set (Lea‐Cox et al., 2001). Studying N dynamics in shoots and across yield levels, demonstrated that up to ~70–80% of absorbed N, P, and K is allocated to fruits, with strong positive correlations between new-shoot biomass, nutrient concentration n, and fruit yield (Fan et al., 2020). Comparative study of NUE in lemon (*Citrus limon* (L.) Burm. f.) versus sweet orange [*Citrus sinensis* (L.) Osbeck] showed species-level differences in biomass partitioning. Lemons invested proportionally more biomass in leaves and less in roots, ensuring higher NUE over sweet oranges. Similarly, lemons presented lower N concentration than sweet orange trees, the former exhibited better photosynthetic-NUE (55–120 mmol CO_2_ g N^-1^ day^-1^) compared to sweet orange (31–68 mmol CO_2_ g N^-1^ day^-1^) (Dovis et al., 2020).

Ca-partitioning studies on response of ^44^Ca labeling in young ‘Salustiana’ sweet orange (*Citrus sineneis* (L.) Osbeck) trees showed Ca taken up from fertilizer accumulated in new flush leaves, twigs, and fibrous roots, mostly bound as insoluble Ca-pectate (Fraction II) in tissues (Morales et al., 2023). Partitioning of Fe treatment with ^57^Fe and ^58^Fe studies on 4-year-old clementine trees (*Citrus clementina* Hort ex Tan) revealed that >90% of total Fe is retained in roots, but fertilizer-derived Fe is preferentially allocated to flowers and young fruitlets t (Martínez-Cuenca et al., 2021). These studies describe nutrients partitioning through roots, leaves, shoots, and fruits, and changes in their concentration over phenological growth phases. A synthesized ‘lifetime allocation strategy’ based on source-sink relationship and nutrient partitioning is still missing.

## Climate change stressors and citrus nutrition

Climate change-related stresses influence citrus nutrition through changes operating at soil, root, canopy, and fruits. These stresses regulate crop phenology, nutrient mobility, root activity, and carbon–nutrient balance, in shaping both tree’s nutritional status and fruit nutritional quality (Medda et al., 2022; Naeem et al., 2024). Such outcomes shed light on shortcomings of traditional fertilization programs in sustaining the current trends of citrus production. Various interventions such as constraint-based fertilization, with matching rootstocks, soil health resilience, and climate-linked decision tools are recommended to maintain citrus yield and nutritional quality under climate change scenario.

### Response to rising temperature and heat stress

Rising temperatures accelerate citrus phenology by advancing induction of vegetative flushes, flowering, and fruit development and compressing the periods of peak nutrient demand into shorter windows (Boaretto et al., 2020; Terán et al., 2023). Heat stress during early fruit development stage alters C-allocation, often promoting vegetative growth at the expense of fruit sinks and increasing demand for nutrients like N, Mg, and K during late summer flushes (Nawaz et al., 2021; Mesejo et al., 2024). Under recurrent heat waves, NUE declines due to hormonal signaling (abscisic acid, jasmonic acid, and salicylic acid) shifting plant metabolism more towards survival than plant growth maintenance (Rodríguez-Azorín et al., 2025).

Roots are usually more heat-sensitive than shoots. Temperatures above ~35–40 °C reduce root elongation, damage membrane integrity, and suppress the expression and activity of nutrient transporters, leading to lower uptake rates of N, P, K, Ca, and Mg per unit root length compared to these responses at 20-25^0^C (Mishra et al., 2023). Use of heat-tolerant rootstocks partially mitigate these adverse effects by maintaining the root hydraulic conductivity, vascular integrity, water, and nutrient transport under high temperature (38/28^0^C) conditions (Rehman et al., 2024).

### Response to drought and water scarcity

Varying prevalence of drought and water scarcity affect nutrient uptake by reducing soil moisture, limiting mass flow and diffusion of mobile nutrients, such as NO_3_, K, Mg, and Ca toward roots, even when soil nutrient availability is adequate. Drought-induced stomatal closure further limits photosynthesis and carbohydrate supply, reducing assimilation and translocation of these nutrients (Medda et al., 2022). Thus, common traits of drought tolerance (da Silva Costa et al., 2025) such as increased production of abscisic acid (a long-distance chemical signal from roots to shoots, mediating stomatal closure to reduce water loss), antioxidant defenses, and osmotic adjustments, have been observed in tetraploid Swingle citrumelo (*Citrus paradisi* Macfad. × *Poncirus trifoliata* (L.) Raf.). In addition to ABA, other hormones such as jasmonic acid and salicylic acid also play crucial roles in mediating plant responses to drought, enhancing the antioxidative capacity of plant cells, reducing lipid peroxidation and maintaining membrane integrity under drought stress (Ghassemi-Golezani and Farhangi-Abriz, 2021). However, these advantages often fail to translate into superior drought tolerance in field conditions, where competition for resources and environmental complexities significantly influence plant responses.

Prolonged drought with use of desalinized irrigation water, increases salinity and B-toxicity risks, which exacerbate nutrient imbalances and impair fruit quality (Navarro et al., 2022; Auñón-Calles et al., 2025). Drought coupled with salinity is reported to disrupt important rhizosphere’s microbial processes, including AMF symbiosis, restricting nutrient acquisition, especially P through reduced spore germination, hyphal growth, and root colonization (Zou et al., 2025; Wan et al., 2025; Tan et al., 2026). Essential components of drought adaptation comprise of importance of root system architecture, leaf anatomical modifications, and stress‐responsive transcription factors. While, agronomic innovations such as precision irrigation, soil management techniques, and plant‐microbe interactions are reviewed due to their potential to enhance sustainable water use (Franco-Navarro et al., 2025). The drought tolerance of X639 (*Citrus reshni* Hort ex Tan × *Poncirus trifoliata* L. Raf.) was evidenced by its ability to maintain plant tissue moisture, membrane and chlorophyll stability, and higher photosystem II efficiency. High-throughput imaging techniques have proven effective in rapidly assessing and differentiating drought-tolerant and drought-susceptible citrus rootstocks based on their photosystem- II efficiency, leaf area, and tissue water content during induced drought stress (Morade et al., 2025). Maintaining SMT around −0.035 MPa during the dry season optimizes yield while reducing water use (Rivera-Hernández et al., 2025). These findings will contribute to the selection and development of drought-tolerant citrus rootstocks to improve citrus production under water-limited conditions.

Relating climate change–driven rainfall in form of heavy precipitation with nutrients transformation on the other hand, enhance nutrients leaching (e.g., NO_3_, Ca, Mg, K, Mn, B, and zinc, Zn) beyond the root zone, in many citrus regions characterized by coarse-textured soils in Florida, USA (Kadyampakeni et al., 2018), Hunan province, China (Cao et al., 2021), northwest and northeast India (Srivastava and Patil, 2016; Rymbai et al., 2026), southern Brazil (Pilon et al., 2023), and in southeast Spain (Segovia et al., 2017). In contrast, flooding and waterlogging reduce soil aeration and damage fine roots, further limiting the nutrients uptake (Duan et al., 2020). To optimize plant nutrition during drought stress, nutrient management strategies must be tailored to water-limited conditions. This includes adjusting fertilizer rates, timing, and application methods to improve NUE and minimize losses. Techniques such as use of water-soluble or slow-release fertilizers, biofertilizers, biodegradable mulches, and applying foliar nutrient sprays especially under HLB-presence, can help maintain nutrient availability and support optimal plant development during drought (Asadu et al., 2024; Keteku et al., 2024). Likewise, efficient irrigation strategies also have important roles to play in water limited conditions. Significant variation in physiological traits like stem water potential, net photosynthetic rate, stomatal conductance, and the amount of leaf chlorophyll was observed in relation to different irrigation strategies, highlighting the highest plant water stress, in particular for the partial root-zone drying irrigation system to replace 50% of ETc in sweet orange, cv. ‘Tarocco Rosso VCR’ [*Citrus sinensis* (L.) Osbeck] grafted on Carrizo citrange rootstock [*Poncirus trifoliata* (L.) Raf. × *C. sinensis* (L.) Osbeck] under Mediterranean climate. The WUE values aided by proximal sensing measures in sub-surface drip irrigation system to replace 80% of Etc were similar to the moderate deficit irrigation treatment and more efficient (up to 50%) as compared to control (Stagno et al., 2025).

### Response to elevated atmospheric C0_2_

Elevated CO_2_ does not function alone, usually acts with high temperature, but stimulates citrus growth and carbohydrate accumulation, leading to an increased biomass and starch reserves (Allen and Vu, 2009). However, this growth stimulation frequently results in nutrient dilution, with lower concentrations of N in leaves and fruits (Gojon et al., 2022). Elevated CO_2_ also alters C-N balance by inhibiting NO_3_-uptake and assimilation, necessitating revised N- benchmarks and fertilization strategies under future climate change scenarios. An elevated CO_2_ has a negative impact on key physiological processes of nutrient acquisition and assimilation in C_3_ plants like citrus. The reasons are largely unknown. Climate change stressors hence influence citrus nutrition by reshaping plant physiology-driven absorption, transport, and utilization of nutrients through interconnected plant and soil pathways than as isolated independent factor.

## Climate change and macronutrient nutrition

Transformation and availability of nutrients under climate change scenario with citrus as a test crop is still far little understood (Srivastava, 2024), despite wealth of information accrued through multi-decadal research on citrus nutrition (Srivastava and Hota, 2020). We have attempted below to portray nutrient response of citrus with emphasis on climate change.

### N-nutrition: mechanisms and management options

Fertilizer-N inputs in mature citrus orchards typically range between 150 and 350 kg ha^-1^year^-1^. Leaching losses of N can be high (50–150 kg ha^-1^ year^-1^), especially when N-fertilizer supply is over-optimal (Alva et al., 2006b; Lidon et al., 2013). These N losses contribute to pollution of ground water and surface water bodies, and there is therefore a great need to reduce these losses (Wei et al., 2024), more so under climate change.

Convergence of salinity, recurrent drought, and erratic precipitation-induced moisture deficit suppressing NO_3_-uptake and assimilation due to inhibition of nitrification and reduced N-mineralization, leading to accumulation of NH_4_-N and NH_3_-volatilization. The competition between chloride (Cl^-^) and NO_3_^+^ ions inhibits the activity of key enzymes involved in N-assimilation pathways under salinity stress. On the other hand, drought stress turns NO_3_-transporters (NRTs) downregulated, while salinity stress induces an upregulation of NRTs in roots as an adaptive response to N-deficiency (Alcon-Bou et al., 2025) under limited transpiration and water availability. Unlike salinity, drought promotes higher root-to-shoot growth ratio while comparing N-deficiency with optimum-N, but ultimately limits N-availability for shoot development (Abobatta, 2022). These responses are of much higher order under salinity plus drought combines with high temperature over their responses under individual stress (Hussain et al., 2023)., Rising atmospheric CO_2_ concentration stimulates vegetative growth and carbon sequestration in sour orange (*Citrus aurantium* L.), however, such physiological gains under a changing climate are conditional (Rasera et al., 2023). Climate change is poised to reconfigure N-dynamics in citrus production systems by limiting N-uptake-efficiency, altering the dynamics of different N-forms, and increasing the risk of N-imbalance driven N-losses (**Table 3**). A greater risk of chronic N-deficiency or imbalance as eCO_2_ (equivalent CO_2_) and high temperature reduce plant N-concentration and soil inorganic N-availability, affecting N-uptake compared to either of the two stresses alone (Soares et al., 2019; Qaderi et al., 2025). In citrus orchards, N -deficiency induced low nitrogen-use-efficiency., N-uptake-efficiency, and N-assimilation efficiency, are therefore key causes of yield and quality losses.

**Table 3:** Physiology associated with N-nutrition in citrus exposed to climate change stressors.

Issues	Key findings	References
Sensitivity of N- forms	Citrus (*C. reticulata* Blanco cv. Ponkan) seedlings sensitive to excess NH_4_^+^ under salinity and drought stress, NH_4_^+^-nutrition inhibits growth, N- uptake, PNUE, and induces oxidative stress evident from an increased accumulation of malondialdehyde and lower activities of nitrogen-metabolizing enzymes like glutamine synthetase and glutamate synthetase	Chen et al. (2023)
N-deficiency	Citrus shows adaptive increase in sucrose export and molar ratio of C/N allocation in leaves for improved root growth, but with trade-offs in growth, yield, and quality in sweet orange [*C. sinensis* (L]) Osbeck) orange seedlings.	Huang et al. (2022)
Extra supply of Mg, N, and Mg+N followed by heat exposure for 12-days	Extra N + Mg-supply maintains elevated photosynthetic and transpiration rates and low ratio of apparent electron transport rate per photosynthetic carbon assimilated as adaptive mechanism to N-deficiency in young lemon (*C. limon* Burm. f.*)* trees.	Boaretto et al. (2020)

PNUE stands for physiological nitrogen- use-efficiency defined as yield per unit of N acquired at whole plant level.

Amid these facts, N-fertilization strategies need application of AMF or demand-driven NO_3_^-^ fertilization in split doses (Wu et al., 2017), slow release fertilizers (Alva et al., 2006b), and N-fertigation (Shirgure et al., 2001) to improve citrus growth and performance by reducing the Cl-accumulation as an evidence of osmotic adjustment mechanism under salinity and drought; and counter N-losses through NO_3_-leaching, NH_3_-volatilization, and denitrification under waterlogged conditions. Such management efforts are productive only, if supported with real-time proximal plant-N sensing to maintain yield and reduce N- losses under changing climates (Nasiro and Mohammednur, 2024).

### P-nutrition: mechanisms and management options

The availability of P in soil involves transformation of both inorganic-P, viz., iron-phosphate (Fe-P), aluminum-phosphate (Al-P), and calcium-phosphate (Ca-P) and organic-P fractions, viz., labile -P (readily available-P), moderately labile-P (not readily available-P), and non-labile P (unavailable-P). Such a transformation is influenced by soil type and soil microbial network (Pang et al., 2024). Role of phosphate solubilizing microbes (PSM) is characterized by key biomarkers having ability to produce alkaline and acid phosphatases, encoded by *phoA*, *phoD*, and *phoX* genes, converting organic-P into the forms that are more plant accessible (Yu et al., 2024).

Under climate change scenario, P-limitations of higher order are anticipated in semi-arid than in tropical or subtropical citrus production systems, due to vulnerability of plant accessible P-forms to P-fixation, restricting further the P-diffusion, causing chronic P-deficiency. Combination of high soil temperature, drought, and pH shifting to alkaline range (pH > 7.5–8) reduce P-uptake and partitioning within the crop (Hu et al., 2023). Elevated CO_2_ increases plant growth and P-demand, altering root morphology, composition of root exudates, and rhizosphere microbial diversity to mobilize more P in low-P soils (Guo et al., 2021). In citrus (*C. grandis*), P -deficiency reduces leaf and stem growth, chlorophyll content, CO_2_-assimilation, and impairs electron transport in Photosystem-I/Photosystem-II (PSI/PSII, both found in thylakoid membranes of chloroplast), in addition to triggering oxidative stress (Meng et al., 2021). In humid citrus production system, high temperature-induced soil warming (equivalent to temperature change of 2.1^0^C crossing 600m gradient) can either increase plant P-content by enhancing mineralization and resorption (Lie et al., 2021) or decrease bioavailable-P and microbial- P by enhancing sorption and leaching of P (Tian et al., 2023).

High temperature coupled with altered rainfall (moisture deficit) strongly modifies PSM and phosphatase activity, increasing biological P-cycling, but often constrained by drought or long-term sorption to recalcitrant P-pools (Tian et al., 2023). In citrus orchards, long-term heavy P-fertilization was observed associated with high build-up of soil P (>30–50 mgkg^-1^) and reduced bacterial diversity and network stability, undermining resilient P-cycling under future climate stress (Zeng et al., 2024). Addressing impact of altered P-dynamics, organic management practices, involving the use of compost and biochar have shown significant improvements in plant-available P, partially offsetting climate-induced decline in mobility and accessibility of P (Pereira et al., 2025). As climate stress intensifies, in order to reduce manifestations of P-deficiency (**Table 4**), biological strategies become increasingly central to P-acquisition, like role of AMF-symbioses and PSM, using P-efficient and AMF-dependent citrus rootstocks (Zhou et al., 2020; 2025).

**Table 4:** Response of P-nutrition to key climate drivers in citrus.

Climate constraints	Implications of P-nutrition	References
High temperature, altered rains -induced moisture deficit, and alkaline soil pH	Lowers labile soil P causing an increased sorbed-P, thereby increasing the risk of P- deficiency.	Maharajan et al. (2021)
Frequent drought with high temperature	Curtails diffusion and uptake of P and raises foliar N:P ratio (> 200.0), signaling onset of severe P -imitations in Satsuma mandarin (*C. reticulata* Blanco) as test crop	Xu et al. (2025)
Elevated CO_2_	Increases growth and P-demand, benefits realized only with sufficient bioavailable-P under AMF-inoculation.	Guo et al. (2021)
Soils with high legacy- P (>73.2 mgkg^-1^)	Reduces PSM-diversity with reference to P- cycling by *Acetobaceraceae* and *Beijerinckiaceae*, and further slowing down the pace of P- cycling in Satsuma mandarin (*C. reticulata* Blanco)	Zeng et al. (2024)

Legacy -P represents unassimilated residual fraction of soil -P.

Climate-sensitive regulation of root exudation and action of carotenoids-derived plant hormones) play a decisive role in sustaining P-uptake under P-deficiency conditions (Soliman et al., 2025). Research is further needed on chemical, molecular, microbiological, and physiological issues relating P-nutrition to improve the understanding on how temperature, pH, drought, and elevated CO_2_ affect the availability, acquisition, and transport of P by plants. This will provide useful insights on sustained plant P-demand by increasing the biological and geochemical controls of plant-soil P cycle, which has important implications for C-fixation in P-deficient soils.

### K-nutrition: mechanisms and management options

Importance of K in citrus assumes a much greater significance under climate-related stresses than non-stress conditions, considering the involvement of K in various metabolic functions such as osmotic adjustment, membrane stability, ROS-detoxification (Calleja-Cabrera et al., 2020), regulating stomatal function, enzyme activation, photosynthesis, and metabolites transport, all central to drought and heat tolerance (Alva et al., 2006a; Papadakis et al., 2023b). On the other hand, agronomic response of K takes into account various fruit quality traits like fruit size, peel thickness, juice acidity, and sugar accumulation, even when leaf symptoms of K-deficit are mild (Jiao et al., 2022; Wu et al., 2024b). At the same time, climate-induced salinization disrupts ionic homeostasis by increasing Na/K ratio and reducing K-concentration in citrus leaves and roots, in addition to rootstock-dependent role in K-partitioning (Navarro et al., 2022). Combined deficits of N, P, and K with heat and drought in Carrizo citrange [*C.* (L) Osbeck x *Poncirus trifoliata* (L.) Raf.] caused significant reduction in growth, gas exchange, pigments accumulation, and oxidative damage when ≥3 stresses co-occur, indicating K-shortage amplifying multifactorial stress injury (Rodríguez-Azorín et al., 2025). The interaction effect of climate change and K-nutrition is further summarized (**Table 5**).

**Table 5:** Climate drivers interacting with K- nutrition in citrus.

Climate drivers	Likely impact on K- nutrition	References
Frequent drought	Reduced mass flow of K to roots and increase in K-need to support osmotic adjustment and stomatal control.	Nieves‐Cordones et al. (2018); Soares et al. (2019).
High temperature	Higher transpiration and growth compared to ambient temperature add to increased K- demand, while heat + K-deficiency is highly damaging to photosynthesis.	Papadakis et al. (2023b); Rodríguez-Azorín et al. (2025).
Water scarcity and desalinized water use	Increased K-need for maintaining hydraulic conductivity of roots and mitigating salt damage under Na^+^/Cl^-^ competition or Na-toxicity conditions.	Navarro et al. (2022)

Manifestations of adequate-K in leaves of navel orange (*C. sinensis* Osbeck), include sugar accumulation via enhanced C-flow from source leaves to fruits, sugar metabolism, and sink strength, improving overall fruit quality (Wu et al., 2024b). K-fertilization reduces various peel disorders in Ehime Kashi 34 (*C. nishinoka* × *C. shiranui*, a hybrid cultivar susceptible to easy fruit splitting) fruit firmness ratio of peel to flesh, photosynthetic rate, stomatal conductance, concentration of intercellular CO_2_, and Ca, K, and K concentration in peel and flesh coupled with upregulation in glycoside metabolites (Jiao et al., 2022). While, in lemon (*C. limon* Burm.), K-fertigation (80 ppm K) increases xylem hydraulic conductivity (transport of water through its xylem tissues under a given pressure gradient) and reduces embolism (blockage of xylem vessels by air bubbles, a major survival threat under drought stress) risk under elevated C0_2_ (400–850 ppm), though these benefits collapse under exposure to drought (zero irrigation for 40-days), implying that K cannot fully offset water stress (Wagner et al., 2020). These studies support efficient K-management integrated with fertigation and foliar-fertilization on K-deficient soils for improved yield, nutrient uptake, and WUE to negotiate high temperature (>33-40^0^C)-induced water stress (Quaggio et al., 2019; Ahmad et al., 2022; Awad et al., 2024).

### Ca-nutrition: mechanisms and management options

Ca, a secondary macronutrient, serves as a second messenger in many developmental and physiological processes, including the response of plants to biotic stress (Thor, 2019). The functional importance of Ca in citrus increases manifolds with exposure to climate change-related stress by reinforcing its role in membrane stability and protection against oxidative damage (Calleja-Cabrera et al., 2020). At the same time, progressive soil and irrigation water salinization associated with climate change elevates Na^+^/Ca²^+^ ratio and increases the risk of Ca-related disorders, influencing both fruit yield and fruit quality (Navarro et al., 2022). In mandarin (*C. reticulata* Blanco) orchards of semiarid region, long-term irrigation with saline-reclaimed water impaired canopy growth, yield, and fruit quality through Cl-accumulation in leaves compared with freshwater (Auñón-Calles et al., 2025). Many citrus peel disorders like cracking, splitting, and albedo breakdown, are closely linked to Ca-deficit or Ca mis-partitioning, even when total leaf Ca is sufficient (Bonomelli et al., 2022). Tracer studies in young citrus trees show Ca-fertilizer enhances biomass, and newly absorbed Ca is allocated mainly to new flush leaves and fibrous roots and bound to cell-wall pectates as structural-Ca (Morales et al., 2023; Dong et al., 2025). Climate-induced changes in growth patterns and transpiration streams therefore reshape Ca-distribution in plant parts. The key roles of Ca and climate stress-related responses of citrus are further highlighted (**Table 6**). Ca-nutrition in relation to climate change hence becomes increasingly challenging with respect to cellular integrity in citrus fruits, especially under desalinized irrigation or water-limited production system.

**Table 6:** Key roles of Ca and climate-linked responses of citrus.

Attributes	Response of Ca	Climate-relevant responses	References
Fruit cracking	Ca or Ca+Si-sprays at 60–90 DAF increase peel-Ca, reduce PG activity, WSP, boost antioxidant defense, and lower cracking in cracking-prone cultivar like Okitsu no 58 (*C. reticulata* Blanco).	More erratic rain and heat-spikes (rapid fruit swelling) increase risks of cracking compared to normal rainfall (total annual rainfall 1117.3 mm) and temperature (average annual temperature 16.9°C), thereby raising the importance of Ca-sprays.	Wang et al. (2024)
Sugar accumulation	Improvement in Ca-responsive *CsMYB36–CsSWEET17* regulatory genes mediating sucrose accumulation in fruits, thereby regulating the fruit development and quality formation.	High temperature (>35^0^C) alters sugar–acid balance by weakening the Ca-signaling involved in sugar loading.	Sheng et al. (2025)
Ca-fertilization response	Ca-fertilization improves growth, even in Ca-rich calcareous soils, with fertilizer-derived Ca predominantly deposited in young tissues.	Maintaining adequate plant available Ca is essential for development of new flush and fruitlets under stress-altered transpiration.	Soares et al. (2019); Morales et al. (2023).

Si, silicon; DAF, days after flowering; PG, polygalacturonase; and WSP, water soluble pectin.

### Mg-nutrition: mechanisms and management options

Mg as a part of chlorophyll molecule, is central to series of metabolic processes like photosynthetic electron transport, CO_2_-assimilation, carbohydrate translocation, and enzyme activation (Ishfaq et al., 2022), besides participating in organic acid, amino acid, and lipid metabolism (Liu et al., 2022). While, under heat and drought stress along with high temperature, fundamental roles of Mg in membrane stability, osmotic regulation, and antioxidant defense are compromised. But, under optimum growing conditions, Mg offers a protection against oxidative damage due to ROS accumulation (Calleja-Cabrera et al., 2020). High temperature coupled with elevated CO_2_ reduces Mg- concentration in plants due to lower Mg-availability (Ishfaq et al., 2022). In major citrus areas of southwest China, Mg-deficiency in >70% of orchard soils and >90% of leaves, were observed largely due to acidification, heavy rainfall, and overuse of NPK fertilizers (Wang et al., 2022).

Different citrus species/cultivars of sweet orange (*C. sinensis* (L.) Osbeck) showed varying sensitivity to photosynthesis and oxidative damage on account of Mg-shortage, so Mg-efficient genotypes/rootstocks play an important role in moderating climate -related stresses (Papadakis et al., 2023a). In young lemon (*C. limon* (L.) Burm. f.) plants, extra Mg-supply along with N improved the tolerance to combined effect of high radiation and temperature, maintained photosynthesis and transpiration, and enhanced the synthesis of antioxidant enzymes for reduced oxidative damage (Boaretto et al., 2020; Ahmed et al., 2023). Meta-analysis across species (including citrus) showed adequate Mg-mediated increase in net CO_2_-assimilation by ~140% and biomass by ~60% over Mg-deficient plants (Hauer-Jákli and Tränkner, 2019). The key yield- and quality-related responses of citrus to Mg- nutrition, together with their relevance under future climate scenarios are further summarized (**Table 7**). Considering acid soils of citrus orchards known for leaching of Mg, applications of Mg-fertilizers or Mg-fortified soil conditioner are vital to sustain soil Mg-balance, high fruit yield, and fruit quality in citrus production systems of humid subtropical regions (Wang et al., 2024).While, Mg-nutrition in calcareous soils (pH >7.0) is often constrained by high Ca levels and high pH, which restrict Mg-bioavailability despite high Mg content (Mousavi et al., 2024).

**Table 7:** Climate-linked responses of citrus to Mg-fertilization.

Attributes	Response functions	Climatic relevance	References
Fruit yield	Mg-fertilization increases navel orange [*C. sinensis* (L.] Osbeck) yield by ~18-28% over no Mg-treatment.	Heat and drought stress together pose greater challenges than either of two stresses alone to sustain photosynthetic capacity for canopy growth.	Hauer-Jákli and Tränkner (2019); Boaretto et al. (2020)
Brix and peel color of fruits	MgO (142-177gplant^-1^)- fertilization increases pulp sucrose by up to ~30% and improves peel coloration coupled with early fruit ripening in ‘Nehwall’ Navel oranges [*C. sinensis* (L.) Osbeck]	High temperature alters sugar–acid balance, while Mg-supply helps maintain this fruit quality trait coupled with fruit color deciding market acceptability	Liu et al. (2022)
Multiple stress tolerance	Supply of Mg or Mg-oxide nanoparticles aid in regulating methylglyoxal (a cytotoxic byproduct generated during abiotic stress or sugar development process in fruits) metabolism, reduces oxidative damage to cell membrane, and improves Mg-uptake-efficiency compared to suboptimum Mg-supply in sweet oranges [*C. sinensis* (L.) Osbeck] under drought, heat, and soil moisture deficit stress.	Mg-sufficient trees develop tolerance against frequent heat waves and combined stresses.	Cai et al. (2019); Ahmed et al. (2023)

In summary, climate change is expected to alter macronutrient dynamics in citrus agroecosystems by exerting simultaneous limitations on transformation, availability, uptake efficiency, and internal allocation. Management of N- and P-deficits will pose challenges under rising temperatures, rainfall patterns deviated from normal, and elevated CO_2_, by reducing nutrient availability while increasing plant demand. These conditions increase the risk of chronic nutrient imbalances, necessitating the adoption of 4R-based fertilization strategies combined with proven rootstock–scion combination. Concurrently, drought, heat, and salinity stresses increase the functional importance of nutrients like K, Ca, and Mg in maintaining physiological processes such as photosynthesis, soil-plant water relations, and membrane stability, while making their management more site-specific. Emerging approaches, including the use of AMF, biochar, composts, and PSMs, offer promising alternatives to enhance NUE and soil health resilience under changing climatic conditions.

## Climate change and micronutrient nutrition

Micronutrient nutrition in citrus is a major focus of research worldwide, particularly in relation to its role in plant defense (Tripathi et al., 2022). This perspective has further expanded to encompass soil–plant health continuum (Srivastava et al., 2022) and rhizosphere microbiome research aimed at developing disease-suppressive soils (Srivastava et al., 2025). However quantitatively, linking these processes to climate change remains a major research challenge.

### Fe-nutrition: mechanisms and management options

Widespread Fe-deficiency induced Fe-chlorosis (often called lime-induced chlorosis featuring interveinal chlorosis) in alkaline and calcareous soils causing heavy loss to fruit yield and quality with poorly colored peel, is a global phenomenon. Climate change is expected to add complexity to transformation and availability of Fe due to rise in soil pH, redox potential and salinity, and drought helped by atmospheric CO_2_-induced high temperature. Fe-nutrition is a formidable challenge under salinity and calcareousness of soils (Soares et al., 2019; Navarro et al., 2022). Waterlogging due to heavy rain or poor drainage down-regulates root H^+^-ATPase (HA1) and ferric-chelate reductase genes (FRO2) by >65% over optimum soil moisture supply level, cutting Fe-uptake and onward translocation of Fe to shoots by ~30–70% (Martínez-Cuenca et al., 2015). As a part of genetic adaptation against Fe-chlorosis, rootstocks differ widely in tolerance to Fe-deficiency, physiological traits such as high Fe/Cu ratio, root ferric-chelate reductase activity, and efficient Fe-translocation to shoots are linked to growth performance (Pestana et al., 2023). Fe-efficient rootstock (e.g., Zhique, *C. wilsonii* Tanaka^)^ maintains higher Fe^2+^ concentration in roots and leaves and up-regulates Fe-acquisition genes (HA1; FRO2; IRT, Fe-regulated transporter; and NRAMP, natural resistance-associated macrophage proteins) for improving Fe-tolerance on high pH, calcareous, and low-Fe soils (Fan et al., 2022). Rootstocks tolerance to lime-induced Fe-chlorosis varies, with trifoliate orange (*P. trifoliata* (L.) Raf.) being very susceptible, while sour orange (*C. aurantium* L.), various mandarins (*C. reticulata* Blanco and *C. nobilis* Lour.), limes (*C. limonia* Osbeck), and rough lemons (*C. jambhiri* Tanaka) are more tolerant than trifoliate orange (Pestana et al., 2005). Hence, climate-induced changes are likely to increase both, the frequency and severity of Fe-related disorders in citrus with combined effect of salinity, drought, and high temperature, underscoring the growing importance of Fe-efficient rootstocks (having specialized root systems that improve Fe-uptake in high-pH or calcareous soils, preventing chlorosis) to sustain productivity under future climatic scenarios. The major climate drivers affecting Fe-nutrition in citrus, the associated physiological constraints, and management strategies are further summarized (**Table 8**).

**Table 8:** Climate drivers, Fe-limitations, and citrus responses.

Climate driver types	Traits of Fe-nutrition	Matching strategies	References
Alkaline and saline, coastal soils.	Lower Fe availability, severe chlorosis, and poor fruit quality.	Use of acidifying glass matrix-based organo-mineral fertilizer, chelated-Fe fertilizers depending upon soil or foliar fertilization and climate driver like salinity and drought and Fe-efficient rootstocks.	Torrisi et al. (2015); Ahmad et al. (2022); Zang et al. (2023)
Frequent waterlogging/flooding creating soil saturation within rootzone.	Suppress Strategy- I (reduction of Fe^3+^ is an obligatory step in making Fe available to plant) responses, reduced Fe-uptake, and distorted Fe- distribution causing localized Fe-deficiency in growing tissues*.	Improved drainage, Fe-tolerant rootstocks, and synchronize the timing of Fe-fertilization avoiding period of anoxia.	Pestana et al. (2005); Martínez-Cuenca et al. (2015)
CO_2_-induced high temperature (> 35–38 °C)	Dilution of Fe in fruits and elevated Fe-requirement.	Optimize Fe-chelate treatment scheduling**, and monitor consequent changes in rhizosphere microbiome composition using Proline-2′-deoxymugineic acid (a phytosiderophore analog)***.	Huan et al. (2012), Gong et al. (2026)

*situation often termed as Fe-inactivation, occurs when Fe is trapped in an unavailable form, such as in root apoplast, preventing it from reaching chloroplast for chlorophyll synthesis.

**Zn and Mn deficiencies became the new limiting factors after Fe-chlorosis correction.

***associated with recruitment of growth promoting microbes such as *Pseudomonas* and *Nitrospira* enriching microbial carbon fixation pathways.

### Copper-nutrition: mechanisms and management options

Cu-deficiency is a widespread nutritional disorder in alkaline or organic matter rich acid soils, while Cu-excess can inhibit plant photosynthesis and induce cellular oxidative stress (Xu et al., 2024). Hence, citrus is highly sensitive to Cu-nutrition and to climate-driven stressors, developing linkages with metabolic functioning of nutrients like high N-induced Cu-deficiency (Mattos Junior et al., 2010) or high Fe-induced Cu-deficiency (Srivastava and Singh, 2005a). Climate-related nutrient stress, particularly under N- and P-limitations due to salinity and drought, influence Cu-acquisition indirectly through strigolactone-mediated changes in root architecture and rhizosphere microbial communities, highlighting the role of climate-sensitive root-inhabited microbes in transformation and availability of Cu for elevated citrus performance (Soliman et al., 2025). The ongoing pace of climate change is likely to intensify both Cu -deficiency in newly established citrus orchards on alkaline soils and Cu- toxicity due to long heavy use of Cu-fungicides in older orchards on acidic soils (Hussain et al., 2023). These studies also highlight the complexity in Cu- management in citrus production under future environmental conditions (**Table 9**).

**Table 9:** Climate-related stresses in Cu- toxicity and -deficiency risks in citrus.

Climate attributes	Cu-related issues	Key Cu–nutrition strategies	References
High temperature, humidity, and high soil borne disease pressure at soil saturation and soil acidification	Chronic Cu- toxicity in old orchards	Limit Cu-fungicide load, keep soil pH ≥6.0–6.5 with liming, add organic matter or humic substances to complex Cu, use Cu-tolerant rootstocks, and periodically monitor leaf and soil Cu level.	Zhang et al. (2024)
Sandy alkaline soils in newly established orchards with low fungicide use	Cu-deficiency causing weak canopy growth and poor root lignification.*	Soil- or foliar-Cu fertilization, balancing with N and Ca, avoiding over-correction for soil pH.	Soares et al. (2019); Huang et al. (2025)
Drought + heat + Cu buildup in soil	Oxidative stress at given toxic level of soil Cu	Root-induced alkalinization and Cu-stimulated release of exudates complex cuprous (Cu^2+^) ions to reduce Cu-solubility for restricting Cu-transport to shoots, causing reduced Cu-uptake and Cu-phytotoxicity.	Chen et al. (2022)

*deposition of lignin in root cell walls, crucial for water transport and developing barrier against pathogen invasion.

While comparing the response of citrus under Cu-deficiency versus Cu-toxicity conditions, some interesting facts emerge. Cu-excess in citrus seedlings causes fibrous root rot, reduced water and nutrients uptake, oxidative stress, photosynthesis decline, and impaired fruit yield along with quality (Giannakoula et al., 2021; Huang et al., 2025). Field studies further support high soil-Cu with leaf -Cu to misshapen and small fruits, indicating a narrow “optimal” Cu-window under rising load of Cu in soils (Mo et al., 2022). These findings provide useful insights about the differential response of Citrus species/cultivars to Cu- tolerance via a series of processes enumerated as root sequestration (process where plants transfer atmospheric C0_2_ captured via photosynthesis, into the soil through their roots, converting it into stable organic matter), cell-wall binding, vacuolar compartmentation, and enhanced synthesis of antioxidants and chelators viz., glutathiones, phytocchelatins, phenolics, and lignins (Wu et al., 2021; Zhang et al., 2024). Climate-driven Cu build-up will make such biochemical traits increasingly important as renewed challenges to address them effectively. These challenges will be easier to address with understanding on physiological significance of Cu-uptake and compartmentalization within plants parts, besides the importance of Cu in cellular metabolic processes.

Managing Cu with regard to citrus performance, optimum available supply of Ca and N aid in balancing Cu-deficiency (Hippler et al., 2018) with foliar fertilization of Cu based either mineral fertilizers or chelates, more so in presence of HLB with soil pH buffered around 6.0 (Hamido et al., 2019).

### Zn-nutrition: mechanisms and management

Zn is one of the extensively researched micronutrients, anchored between plant nutrition-driven production and plant defense. Zn-deficiency in citrus is reported worldwide (Srivastava and Singh, 2005b) and usually characterized by interveinal chlorosis, small little leaves (mottle leaf), resetting of leaves, coupled with poor flowering and fruit set, frequently implicated with citrus decline/blight (Srivastava and Singh, 2004; Wang et al., 2022). While, optimum Zn improves plant tolerance-related traits like water relations, membrane integrity, and antioxidant enzymes (Xie et al., 2025). An elevated CO_2_ coupled with high temperature reduces Zn -availability in human diets by inducing ‘Zn penalty’ in crops, defined as increase in crop yield with poor nutritional quality due to Zn-fertilization (Soares et al., 2019). Although, citrus-specific CO_2_–Zn nutrition response data are lacking. The similar mechanisms viz., Zn dilution effect on nutritional quality of citrus, alteration in crop phenology, and changes in soil biology are expected to influence absorption, transport, and utilization efficiency of Zn under climate-driven salinization, erosion, and intensified fertilization.

Linking availability of Zn to climate change, altered rainfall modifying soil moisture, and redox dynamics (Ahmad et al., 2022), lead to variation in Zn-solubility and frequently exacerbating Zn-deficiency in calcareous citrus soils facing depleted native Zn-supply (Navarro et al., 2022). As climate variability intensifies, these soil-driven constraints increase the incidence and severity of Zn-related nutritional disorders in citrus. These multiple constraints therefore add to the importance of targeted Zn-management strategies to sustain tree vigor and productivity under future climate scenarios (**Table 10**). Use of Zn-efficient rootstocks (capacity of rootstock to acquire Zn from the soil, selected through screening methods, such as hydroponic cultures or soil-based pot/field trials) is one of the important cultural interventions in managing Zn-constraints. A Zn-efficient rootstock, *C. junos* (known as Yuzu, a hybrid of sour mandarin, *C. reticulata* Blanco var. Austera and Ichang papeda, *C. ichangensis* L.) increased Zn concentration in all organs by 46–98% and improved the plant growth and C–N metabolism compared with trifoliate orange (*P. trifoliata* (L.) Raf.) (Wang et al., 2022). Climate-driven menace of alkalinity and salinity, degrading soil fertility will further magnify genotype differences to Zn-response. Zn-fertilizer source and soil texture further define the magnitude of crop response to Zn-fertilization. For example, a greater magnitude of Zn-fertilization (18g Zn plant^-1^ through stable ^68^Zn) response was observed on plant dry biomass and Zn-availability on sandy loam soil (18% clay) compared to clay soil (64% clay) in sweet orange (*C. sinensis* (L.) Osbeck, cv Tobias) grafted on Sunki mandarin (*C. sunki* hort. ex. Tan.) (Hippler et al., 2015). Long-term field trials showed rise in leaf Zn-content, increased cumulative yield by ~20%, reduced ROS-accumulation, and elevated catalase activity in mandarin (*C. reticulata* Blanco) and sweet orange (*C. sinensis* (L.) Osbeck) on account of fertigation with Zn-EDTA (Zn-chelate) or foliar fertilization with ZnNO_3_ (zinc nitrate) or ZnSO_4_ (zinc sulphate) (Boaretto et al., 2023).These studies warrant at addressing the narrow gap between Zn-essentiality and Zn-toxicity in relation to nutritional quality of citrus fruits, supported with underlying mechanisms involved at molecular, physiological, and agronomical levels.

**Table 10:** Key climatic pathways facilitating improved Zn-nutrition in citrus.

Climate change issues	Zn-related problems	Important interventions	References
Alkaline, calcareous soils, with low soil organic matter	Zn -fixation driven expression of Zn- deficiency, despite Zn-fertilization	Foliar sprays of chelated -Zn (e.g., Zn-EDTA) or mineral Zn-fertilizers (e.g., ZnO, ZnSO_4_), fertigation of Zn-fertilizers or Zn-chelates, and treatment of organic fertilizers (e.g, Zn-lignosulfonates).	Hippler et al. (2015); Bhatana et al. (2022)
High temperature, drought, and rain-induced soil moisture deficit	Oxidative stress imparting reduced fruit set coupled with poor fruit quality attributes	Maintain adequate Zn to support the synthesis of Cu- and Zn-SOD, stress enzymes, avoiding either Zn-deficiency or Zn-toxicity	Fu et al. (2016); Wang et al. (2022)
Stable fruit yield and quality	Zn-deficiency linked to citrus decline, poor fruitlets retention, and reduced juice content	Repeated foliar Zn sprays, incorporating K and growth regulators (e.g., IAA or GA_3_), improves fruit retention and juice quality parameters, besides fruit yield	Srivastava and Singh (2005b); Arshad et al. (2024)

Zn-EDTA, ZnO, ZnSO_4_, SOD, IAA, and GA_3_ stand for Zn-ethylene diamine penta-acetic acid, zinc oxide, zinc sulphate, superoxide dismutase, indole acetic acid, and gibberellic acid, respectively. Zinc nano fertilizers and Zn-solubilizing bacteria are also used for meeting Zn-requirement of citrus.

### Mn-nutrition: mechanisms and management

Mn is one of the important micronutrients, displaying high sensitivity of citrus to suboptimum Mn-supply level (Tutmez et al., 2009). Climate-driven nutrient stress, with N- and P-limitations, modifies Mn-acquisition indirectly through strigolactone-mediated changes in root architecture and rhizosphere microbial communities (Soliman et al., 2025). While, high rainfall and soil saturation moisture increase redox change induced Mn-solubility (Mn-oxides with variable Mn valencies, Mn^3+^ and Mn^4+^ reduced to Mn^2+^, significantly increasing its mobility and plant availability), increasing the risk of Mn-toxicity in waterlogged acidic soils, and exacerbating Mn-deficiency in arid and alkaline soil environments (Navarro et al., 2022; Zhang et al., 2024). Mn-toxicity versus Mn-deficiency in citrus under changing climatic conditions revealed some interesting facts. In sour pummelo[(*C. maxima* (Burm) Merr.], high Mn at low pH (3.0) was associated with severe leaf necrosis, interveinal chlorosis, impaired chloroplast development, and low rate of photosynthesis. Wu et al. (2024a) observed low soil pH contributes to increasing fruit acidity in lemon (*Citrus lemon* L.), partially by inhibiting citric acid degradation due to the reduced cystolic-Fe (Cyt-Fe) concentrations. While, raising pH from 3.0 to 5.0 in soils of Sour pummelo (*C. maxima* (Burm) Merr.) was associated with: i. reduced Mn-toxicity, ii. reduced Mn-uptake, iii. maintenance of nutrient homeostasis under Mn-stress, iv. reducing Mn excess-induced impairment of thylakoid structure and phosphoenolpyruvate carboxylase, and inhibition of chlorophyll biosynthesis, and v. increasing C0_2_ assimilation rate and subsequent seedling growth under Mn-excess (Rao et al., 2025).

Subsequent studies (Rao et al., 2026) provided new evidence on the roles of ROS and methylglyoxal detoxification systems in the augmented pH-mediated amelioration of oxidative damage in Sour pummelo leaves and roots caused by Mn-excess, as well as a basis for correcting Mn-toxicity by augmenting soil pH. Excess-Mn also induced secondary Fe- and Mg-deficiencies mediated oxidative stress (Fernando and Lynch, 2015). On the contrary, in alkaline and calcareous citrus soils, Mn is less available manifesting reduced photosynthesis, lignin formation, fruit yield, and fruit size with firmness (Atta et al., 2021; Kwakye and Kadyampakeni, 2022). Other climate stresses like salinity, drought, and high temperature transform different pools of soil-Mn by promoting Mn-oxidation into Mn-oxides and reducing Mn-bioavailability. However, these conditions can also increase microbial decomposition of organic matter, which can temporarily release organic-bound Mn into readily available pool of soil -Mn (Zhang et al., 2024).

In major citrus regions of China, widespread soil acidification has produced low pH and high available Mn, with ~50% of soils and leaves showing Mn concentration above sufficiency range, indicating frequent Mn-excess in citrus orchards (Wu et al., 2022). A broad survey of 503 orchards in southeast China showed large spatial variation, ~21% of soils registered excessive Mn content (>50 mg kg^-^¹) under soil pH conditions (Xu et al., 2025). These climate-induced changes are expected to make Mn- nutrition, difficult to manage in citrus. The importance of site-specific management strategies meeting Mn-requirement of crop through soil -or foliar fertilization and rootstock intervention seem crucial in ensuring optimum Mn-nutrition under climate change (**Table 11**).

**Table 11:** Climate-driven changes in Mn- availability of soil and crop response of citrus.

Climatic conditions	Mn-nutrition related issues	Key strategies	References
More acidic, waterlogged, and humid soils	Mn- toxicity evident from - leaf necrosis and nutrient imbalances	Raise pH with lime, improve drainage, avoid use of Mn-fertilizers, and monitor leaf Mn level with suitable rootstock	Papadakis et al. (2006); Khan et al. (2020)
Alkaline, arid, and calcareous soils	Chronic Mn-deficiency and poor stress tolerance	Foliar Mn-sprays or soil application of MnO (9 kgha^-1^) or MnSO_4_ (11.8 kgha^-1^) and treatment with organic composts	Wang et al. (2016); Uthman et al. (2022)
Climatic variability characterized by high temperature, moisture deficit, and HLB pressure	Fluctuating Mn status coupled with oxidative stress	Split Mn-application (soil + foliar, MnSO_4_ at 8.9-11.5 kgha^-1^), keeping Mn within optimal range to support Mn-SOD synthesis and improve photosynthesis for prolong productive life of HLB-infected citrus trees	Kwakye et al. (2020); Atta et al. (2023)

### B-nutrition: mechanisms and management options

B is a kind of micronutrient which has a limited phloem mobility and redistribution, thereby interfering with B-use-efficiency, leading to leaf vein corking, leaf yellowing, reduced root elongation, and lower fruit quality (e.g., small, hard, and misshapen fruits). High temperature, drought, and salinity frequently co-occur with B-stress under climate change, that are more damaging than individual stress (García-Sánchez et al., 2020; Navarro et al., 2022). B-stress affects photosynthesis, nutrient uptake, organic acid, sugar metabolism, and development of antioxidant systems in citrus (Ferreira et al., 2020; Yang et al., 2023). Increasing drought frequency and the expanded use of saline irrigation water under arid conditions promote B- accumulation in plant tissues, elevating the risk of B-toxicity and impairments in fruit set, fruit development, and quality (Luo et al., 2024; Auñón-Calles et al., 2025). In arid and semiarid citrus regions, climate-driven water shortages are promoting the use of desalinated seawater and other non-conventional sources that often contain high B, Na, and Cl, increasing B- toxicity risk in sensitive citrus species (Aparicio-Durán et al., 2021b). Hence, climate-induced increase in soil moisture, low pH, and leaching dynamics accentuate B-deficiency in certain citrus production systems, emphasizing the dual risk of deficiency and toxicity in an overlapping manner. Therefore, the ongoing climate change is likely to amplify the complexity with B -nutrition in citrus.

For example, B mitigates drought stress by enhancing the activity of antioxidant enzymes such as SOD and CAT, which scavenge ROS and reduce oxidative damage (Aydin et al., 2019). This regulation of oxidative stress contributes to cellular homeostasis, helping plants maintain growth and metabolic activity even under limited water availability. B regulates aquaporins, proteins responsible for facilitating water movement across cell membranes, which enhance water uptake and distribution in plants, facilitating salinity-boron stress adaptation mechanisms (Nicolas-Espinosa et al., 2024). Furthermore, B interacts with phyto-hormones such as ABA to modulate stomatal behavior, promoting stomatal closure to minimize water loss through transpiration. This multifaceted role of B in drought tolerance underscores its importance in maintaining cellular function and supporting plant survival during water stress (Qu et al., 2024). Increasing the need for precise water management and site-specific B-fertilization strategies are foremost important to maintain productivity and fruit quality (**Table 12**). The response of rootstocks in addressing dual B-risks aid in developing matching management strategies (Mattos Junior et al., 2017; Zhou et al., 2020) and moderating the impact of HLB on performance of citrus orchards (Dong et al., 2021).

**Table 12:** Response of citrus cultivars and rootstocks addressing B-stress.

Factors	Key findings	References
Scion cultivar	Citrus scion varieties differ widely in B-deficiency or B-toxicity tolerance; and responses involve primary metabolism associated with changes in organic acids and amino acids.	Navarro-Pérez et al. (2024)
Rootstock options	Modulate B-uptake, transport, anatomical changes, and gas exchange, shaping tolerance to both B-deficiency and B-toxicity.	Mesquita et al. (2016), Ferreira et al. (2020)
New tolerant rootstocks	Novel rootstocks (e.g., UFR-6, 2247×6070-02-2, Forner-Alcaide 5, Swingle citrumelo as hybrid of *C. paradisi* L. X *C. trifoliata* L.) show improved tolerance to B-toxicity via lowered transpiration, controlled B-transport, and activated antioxidant systems, characterized by ascorbate peroxidase, SODs, and quaternary ammonium products.	Simon-Grao et al. (2018); Navarro-Gochicoa et al. (2024)

Thus, climate change is expected to influence micronutrient nutrition in ways that increase the simultaneous recurrence of deficiency and toxicity risks. Across micronutrients, CO_2_-driven nutrient dilution will intensify Fe-chlorosis, expand dual Cu-toxicity and -deficiency related constraints, Zn-limitation in degraded and alkaline soils, and destabilize Mn-availability through redox- and moisture-sensitive pathways. B- nutrition becomes more vulnerable, as climate change compresses its narrow safe range, increasing deficiency via leaching and acidification, while elevating toxicity risks under use of desalinized or B-rich irrigation waters. Although, experiments addressing climate × cultivar interaction, are limited for most of the micronutrients, tolerance is increasingly governed by ion exclusion, compartmentalization, and efficient uptake under multiple stress conditions. Sustaining citrus productivity, fruit quality, and nutritional value under future climates will therefore depend on integrated micronutrient-specific management strategies. Though, we hardly talk about residual effect of micronutrients.

Expanding on economic feasibility, adaptation barriers, and infrastructure needs, especially in water-limited and resource-constrained regions, would enhance practical utility of such efforts. Therefore, citrus nutrition in water-limited environments requires a policy change from traditional soil-based fertilization to efficient, water-saving strategies like fertigation using subsurface fertigation combined with sensors (Meshram et al., 2025) and redistributing the nutrient types according to phenology of a given rootstock-scion combination. Economic feasibility in terms of cost of adoption, return on investment, and operational savings on fertigation need further revisit (Panigrahi et al., 2017). While, an analysis on adaptation barriers featuring water savings through use of high-density dwarfing rootstocks consuming less water per unit canopy area; initial capital investment and establishing drip irrigation (Shirgure et al., 2001); soil salinity coupled with water salinity; limited technical knowledge; and risk-averse perception of growers would further help in negotiating with such barriers. Infrastructural needs requiring low volume drip irrigation and micro-sprinklers, monitoring sensors, water storage facility, and mulching materials are equally pivotal to success of such programs as climate resilient options.

## Climate - HLB interaction

Climate variability exposes citrus to temperature extremes and irregular water supply that elevate ROS, depress photosynthesis, and destabilize yield sustainability. In cold-stress experiments with Carrizo citrange (*C.* (L) Osbeck X *P. trifoliata* (L.) Raf.) and Cleopatra mandarin (*Citrus reshni* hort. ex Tanaka), photosynthetic efficiency and gas exchange decline; while lipid peroxidation rises, demonstrating genotype-dependent sensitivity to oxidative damage. Similar crop response occurs under drought and episodic flooding, where biomass accumulation and chlorophyll development are suppressed, but certain HLB-tolerant rootstocks sustain higher soil-plant analysis development and growth under moderate stress, underscoring genetic variation in resilience (Aparicio-Durán et al., 2021a; Vives Peris et al., 2025).

These climate-driven oxidative load are further amplified by presence of HLB. The pathogen, *Candidatus Liberibacter asiaticus* (CLas) infection triggers a chronic immune response in phloem, including ROS overproduction, callose deposition, and immune gene induction, culminating in phloem cell death and transport failure (Ma et al., 2022). This immune-mediated pathology reduces the margin for coping with additional climatic shocks. Anatomical and physiological studies corroborate phloem collapse and obstruct carbohydrate flow in affected tissues (Brodersen et al., 2014), linking ROS-rich immune activation to systemic decline in tree performance. Critically, large fractions of the fine-root system are lost well before canopy symptoms (Graham et al., 2013), shrinking water and nutrient uptake capacity, so that even brief heat or dry spells translate into sharper oxidative spikes at the leaf and phloem level (Shahzad et al., 2020). Field surveys and root mass measurements document ~30–40% presymptomatic fibrous root loss (and higher losses as decline advances), directly tying CLas infection to belowground impairment that predisposes trees to climate stress (Johnson et al., 2014). The centrality of ROS in this interaction is further supported by nano-biotechnology: foliar manganese-oxide nanozymes scavenging ROS by ≈60% in infected trees and rapidly improve pigments and visible symptoms, the evidence that lower oxidative load alter disease expression under fluctuating environments (Li et al., 2025).

Two management levers consistently moderate this feedback loop: i. balanced and elevated nutrition and ii. precise irrigation.

### Balanced nutrition

Multi-site and multi-year studies show that balanced macro- and micronutrients, especially K, Mg, Ca, Zn, Mn, Fe, and B, improve root density, canopy growth, and in several cases, stabilize yield or postharvest quality under endemic HLB (Morgan et al., 2016). Split applications and combined soil plus foliar delivery increase leaf Mn and Zn, leaf area index, and fruit quality indices relative to controls (Atta et al., 2023; Shahzad et al., 2025). Mechanistically, keeping Mn and Fe in optimal ranges supports photosynthesis and antioxidant enzyme systems (e.g., Mn-SOD), directly buffering ROS surges that accompany climate shocks and CLas-triggered immunity. Soil/foliar delivery interactions and micronutrient behavior in sandy Entisols (e.g., differential sorption and leaf uptake of Mn, Zn, B) further warrant for tailored programs by site and season rather than one-size-fits-all recipes (Vashisth, 2020; Kwakye and Kadyampakeni, 2022; Shahzad et al., 2025).

### Irrigation adjustment

Because HLB damages fine roots, frequency and placement of water (not just seasonal totals) are pivotal to avoid transient deficits that spike ROS and accelerate fruit drop (Dong et al., 2021). A two-year field study in Florida showed that more-frequent irrigations with the same total, weekly water (multiple short events per day vs. every-other-day) significantly improved leaf water status, increased fruit set, and produced higher yields in HLB-affected ‘Valencia’ sweet orange—demonstrating that steady root-zone moisture can boost productivity without increasing total water applied (Sutton and Vashisth, 2025). Field and greenhouse studies further showed that ET (Evapo-transpiration) and soil-moisture–guided, frequent-small irrigations at moderate rates maintained water status, enhanced fine-root length density, and sustained canopy growth without excessive leaching in young or high-density plantings. An optimized deficit rates (≈50–80% reference evapotranspiration, Eto or ≈62–81% crop evapotanspiration, ETc) can maintain or improve canopy development and root life span compared with conventional schedules, indicating that controlled deficits and steadier moisture dampen stress oscillations (Kwakye and Kadyampakeni, 2023). Intensive micro-irrigation patterns also concentrate functional roots within wetted zones, providing an advantage when root systems are already compromised by HLB (Kadyampakeni et al., 2014). Finally, irrigation water quality and soil pH matter: mitigating bicarbonate-driven alkalinity via water/soil acidification increased root density, nutrients availability, yield, and soluble solids in HLB-affected groves, underscoring irrigation–nutrition integration as best practice (Morgan and Graham, 2019).

Climate fluctuations and HLB hence converge on a common mechanism—excess ROS and compromised phloem/roots that magnifies stress injury. The most consistent, evidence-based mitigation is a paired program: i. balanced macro/micronutrient supply (with attention to Mn, Zn, Fe, B and site-specific delivery) to bolster photosynthesis and antioxidant capacity; and ii, precise, sensor-guided irrigation (optimized frequency and ET-based rates, coupled with water-quality/pH management) to minimize transient water stress and protect fine-root functioning. Together these interventions reduce oxidative expression and improve growth, quality, and productivity in HLB-affected citrus under an increasingly variable climate. However, the decisive and consequential role of plant nutrition in moderating the HLB impact on citrus performance, is still a debatable subject. Studies across major citrus belts comprising China (Xia et al., 2011), Pakistan (Razi et al., 2011), and Florida, USA and Sao Paulo, Brazil (James et al., 2024) reveal therapeutic utility of plant nutrition with enhanced foliar nutrition as new normal in extending the performance of HLB-affected trees for additional 4–5 years with effective vector control. In the presence of HLB, the concept of site-specific balanced fertilization coupled with nutrients partitioning across growth sages deserves to be revisited afresh with the support of revised leaf nutrient standards, matching with commercial usage of particular rootstock-scion combination. These knowledge gaps will add an element of sustainability in quality production of citrus amid prevailing HLB-incidence and climate change.

## Priority research directions

Nutrient management amid climate change requires moving beyond isolated, short-term, and yield-centered approaches towards integrated, field-oriented research as a major strategic research objective. Future progress in citriculture will depend on placing nutrient management strategies at the core of climate adaptation, to sustain efficiency within the citrus industry, Yang et al. (2024) suggested three-prolonged new technologies aiming at: i. implement a regional coordinated development strategy for citrus fertilizer reduction; ii. intensify policy guidance, publicity, and training efforts related to citrus fertilizer reduction; and iii. Initiate drive for efficiency enhancement and carbon emissions reduction. Such nutrition-centered holistic frameworks are essential in the wake of climate change as static-cum-dynamic issue. Integrating stress physiology aiming at NUE with omics tools like nutriomics-assisted climate proofing and mainstreaming climate change is required. Developing three-tier process-based crops, soil, and water models with fruit quality and nutrition modules will further maximize the outcomes under region-specific climate scenario. As a further follow-up, it is important to establish long-term field-scale trials (> 10-years) evaluating fertigation schedules organic composts, bioinoculants including AMFs for production sustainability, building up passive soil carbon pool, and to stabilize crop demand-driven nutrient-supply. Optimization, recurrent use, and further refinement of 4R-based fertilization with emphasis on NUE and WUE will add a new support tool under climate change scenario. Amid multiple nutrient deficiencies, achieving land degradation neutrality through biochars (biological charcoal) as soil amendment is a time-tested buffer for improved NUE, WUE, and soil carbon sequestration.

Biological properties of soil are more sensitive to climate change than physical or chemical properties. Phytobiome manipulation via changes in structural and functional composition of microbial niches is another important area of research by bioprospecting soil-plant health (Srivastava et al., 2022) with regard to development of microbial consortia and disease suppressive rhizosphere through a process called rhizosphere hybridization (Srivastava et al., 2025). Facilitating endophytes-aided carbon sequestration into the perennial framework of woody citrus plants, will add an additional endurance for biochemical preparedness against climate change. While addressing these issues, role of rootstocks is undeniable in addressing tolerance against drought, heat, and salinity via adjustments in root architecture, ion exclusion, antioxidant regulation, and hormonal signaling. Some important clues are obtained from limited success obtained in this regard:

- Drought/heat: Carrizo citrange (*C. sinensis* (L) Osbeck X *P. trifoliata* (L.) Raf.) improves scion antioxidant system, raffinose, galactinol, and salicylic acid accumulation, enhancing tolerance to combined drought–heat stress (Naeem et al., 2024). While, Bitters (hybrid of Sunki mandarin as *C. sunki* hort.ex.Tan. X Swingle trifoliate orange as *P. trifoliata* (L.) Raf.), Furr (hybrid of Sunki mandarin as *C. sunki* Hort.ex.Tan. or *C. reticulata* Blanco X trifoliate orange as *P. trifoliata* (L.) Raf), X639 (hybrid of *C. reticulata* as Cleopatra X *P. trifoliata* as Rubidoux), and some Rough lemon (*C. jambhiri* Lush) derivatives show strong drought tolerance through development of vigorous root system, WUE, and ROS control (Modica et al., 2025).
- Salinity and high-B water: Sour orange (*C. aurantium* L.), Cleopatra mandarin (*C. reshni* hort.ex Tanaka), and US942 (hybrid of Sunki mandarin as *Citrus reticulata* “Sunki” X Trifoliate orange as *P. trifoliata* “Flying Dragon”) restrict entry of Na^+^/Cl^-^ in roots, reduce leaf margins burning due to salt toxicity; however mechanisms differ among tolerant rootstocks (Moreno-Lora et al., 2025).
- High temperature: Brazilian sour orange (*C. aurantium* L.) and Gadha-dahi (a herbaceous plant as *Boerhavia diffusa* L.) maintain peel color pigments, WUE, and protective anatomy characterized by thicker epidermis and well developed vascular bundles (Rehman et al., 2024).

**Figure 2:**
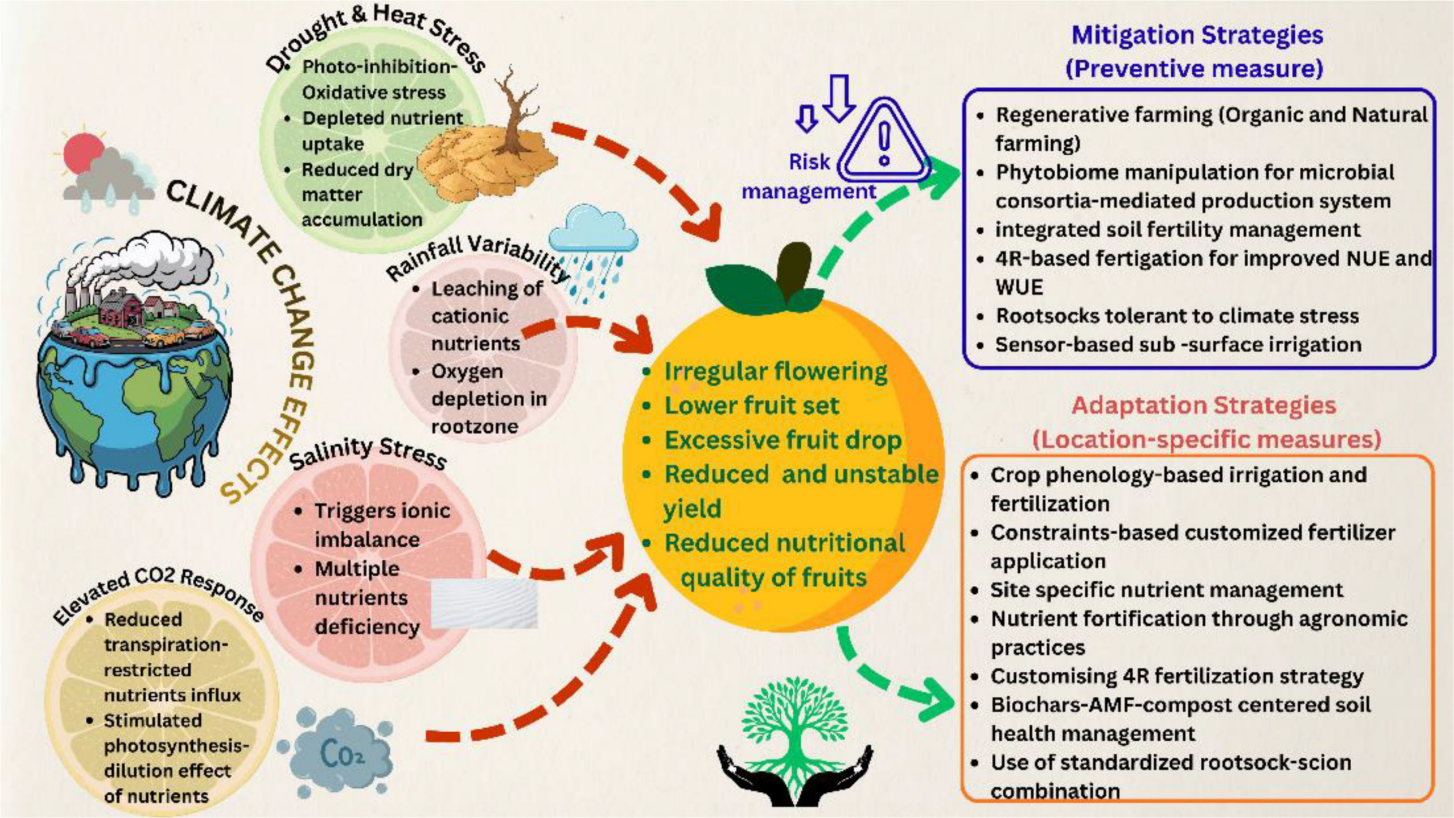
Conceptual framework highlighting different pathways from climate drivers to management interventions (mitigation and adaptation) emphasizing citrus nutrition under climate change scenario.

A conceptual framework (**Figure 2**), summarizing pathways from climate drivers to management interventions affecting citrus performance and mitigation and adaptation strategies (**Figure 2**) offers an integrated tool-kit.

These research priorities need a back-up support by strengthening socio-economic and knowledge-transfer research to improve adoption of climate-smart nutrition practices at farm level. The adaptive capacity and institutional support are limited in many citrus-producing regions. Various adaptation barriers (e.g., financial constraints, human resource, knowledge gaps, institutional issues, lack of awareness or motivation, technical, and infrastructural limitations) and operational barriers (e.g., understanding, planning, and implementation phase) are equally important to obtain the desired success. However, the strongest barrier in non-priority and priority sectors is the limited funding for climate change adaptation, besides lack of participation from local communities (Putri et al., 2025).

## Conclusions

Climate change-related multiple stress is invariably witnessed across citrus production regions, jeopardizing the incentives gained on technology-driven sustainability in quality production. Unfortunately, historic data-based climate modelling for forecasting fruit yield or quality has not been so successful, considering the dynamic nature of climatic stresses. This is where researchers find some limitations in solving the nexus between citrus nutrition and climate change to provide reproducible solutions under varying Future course of climate change will therefore require a shift from static fertilization to climate-smart, monitoring-based nutrition programs, fine-tuned to regional preferences. Finally, closing key knowledge gaps demand long-term, field-scale experiments in tune with multi-stress scenarios, besides development of process-based models linking climate-soil–water–salt continuum, nutrition, and fruit quality to enable region-specific adaptation and decision support. Collectively, a nutrition-centered adaptation framework anchored in diagnostics, soil health, and genetic tolerance collectively offer the most practical strategy to safeguard citrus productivity, fruit quality, and nutritional quality, as climate risks continue intensifying with ominous uncertainty.

## References

[B1] Abbass K. Qasim M. Song H. Murshed M. Mahmood H. Younis I. (2022). A review of the global climate change impacts, adaptation, and sustainable mitigation measures. Environ. Sci. Pollut. Res. Int. 29, 42539–42559. doi: 10.1007/s11356-022-19718-6. PMID: 35378646 PMC8978769

[B2] Abobatta W. F. (2022). Abiotic stress and citriculture. J. Appl. Biotechnol. Bioeng. 9, 138–140. doi: 10.15406/jabb.2022.09.00301

[B3] Ahmed F. El-Rahman B. Mohamed A. (2025). The impact of climate change on citrus production in Sharkia governorate. Fayoum J. Agric. Res. Dev. 39 (3), 566–581. doi: 10.21608/fjard.2025.393015.1128

[B4] Ahmad N. Hussain S. Ali M. Minhas A. Waheed W. Danish S. (2022). Correlation of soil characteristics and citrus leaf nutrients contents in current scenario of Layyah district. Horticulturae 8 (1), 61. doi: 10.3390/horticulturae8010061

[B5] Ahmed N. Zhang B. Bozdar B. Chachar S. Rai M. Li J. (2023). The power of magnesium: unlocking the potential for increased yield, quality, and stress tolerance of horticultural crops. Front. Plant Sci. 14, 1285512. doi: 10.3389/fpls.2023.1285512. PMID: 37941670 PMC10628537

[B6] Alcón-Bou N. Guzmán S. M. Rossi L. (2025). Citrus salinity tolerance in systematic review of cultivars selection trials, grafted versus non-grafted trees, and scion contributions. HortScience 60, 994–1002. doi: 10.21273/HORTSCI18850-25

[B7] Allen L. H. Vu J. C. V. (2009). Carbon dioxide and high temperature effects on growth of young orange trees in a humid, subtropical environment. Agric. For. Meteorol. 149 (5), 820–830. doi: 10.1016/j.agrformet.2008.11.002

[B8] Alva A. Mattos D. Paramasivam S. Patil B. Dou H. Sajwan K. (2006a). Potassium management for optimizing citrus production and quality. Int. J. Fruit Sci. 6 (1), 3–43. doi: 10.1300/j492v06n01-02

[B9] Alva A. Paramasivam S. Fares A. Obreza T. A. Schumann A. W. (2006b). Nitrogen best management practice for citrus trees: II. Nitrogen fate, transport, and components of N budget. Sci. Hortic. 109 (3), 223–233. doi: 10.1016/j.scienta.2006.04.011

[B10] Aparicio-Durán L. Gmitter F. G. Arjona-López J. M. Calero-Velázquez R. Hervalejo Á. Arenas-Arenas F. J. (2021a). Water-stress influences on three new promising HLB-tolerant citrus rootstocks. Horticulturae 7 (10), 336. doi: 10.3390/horticulturae7100336

[B11] Aparicio-Durán L. Gmitter F. Arjona-López J. Grosser J. Calero-Velázquez R. Hervalejo Á. (2021b). Evaluation of three new citrus rootstocks under boron toxicity conditions. Agronomy 11 (12), 2490. doi: 10.3390/agronomy11122490

[B12] Arshad I. Saleem M. Akhtar M. Shani M. Farid G. Jarecki W. (2024). Enhancing fruit retention and juice quality in 'Kinnow' (Citrus reticulata) through the combined foliar application of potassium, zinc, and plant growth regulators. Horticulturae 10 (12), 1245. doi: 10.3390/horticulturae10121245

[B1100] Asadu C. O. Ezema C. A. Ekwueme B. N. Onu C. E. Onoh I. M. Adejoh T. . (2024). Enhanced efficiency fertilizers: Overview of production methods, materials used, nutrients release mechanisms, benefits and considerations. Environ. Poll. Manag. 1, 32–48. doi: 10.1016/j.epm.2024.07.002

[B13] Atta A. A. Kadyampakeni D. M. Morgan K. T. Vashisth T. (2023). Nutrient management impacts on HLB-affected 'Valencia' citrus tree growth, fruit yield, and postharvest fruit quality. HortScience 58 (special issue), 953–961. doi: 10.21273/HORTSCI17110-23

[B14] Atta A. Morgan K. Kadyampakeni D. Mahmoud K. (2022). Spatial and temporal nutrient dynamics and water management of huanglongbing-affected mature citrus trees on Florida sandy soils. Sustainability 14 (12), 7134. doi: 10.3390/su14127134

[B15] Atta A. Morgan K. Kadyampakeni D. Mahmoud K. (2021). The effect of foliar and ground applied essential nutrients on Huanglongbing-affected mature citrus trees. Plants 10 (5), 925. doi: 10.3390/plants10050925. PMID: 34066426 PMC8148103

[B16] Auñón-Calles D. Pinciroli M. Nicolás E. Gil-Izquierdo Á. Gabaldón J. Sánchez-Iglesias M. (2025). Agronomic and metabolic responses of Citrus clementina to long-term irrigation with saline reclaimed water as abiotic factor. Int. J. Mol. Sci. 26 (7), 3450. doi: 10.3390/ijms26073450. PMID: 40244385 PMC11989240

[B17] Awad A. Hussein H. Samad A. Belal H. (2024). Foliar nourishment with different potassium sources to maximize yield through improving nutrient uptake in Citrus aurantifolia trees grown in potassium-deficient soil. J. Soil Sci. Plant Nutr. 24, 7151–7166. doi: 10.1007/s42729-024-02030-2

[B18] Aydin M. Tombuloglu G. Sakcali M. S. Hakim K. R. Tombuloglu H. (2019). Boron alleviates drought stress by enhancing gene expression and antioxidant enzyme activity. J. Soil Sci. Plant Nutr. 19, 545–555. doi: 10.1007/s42729-019-00053-8

[B19] Bacelar E. Pinto T. Anjos R. Morais M. Oliveira I. Vilela A. (2024). Impacts of climate change and mitigation strategies for some abiotic and biotic constraints influencing fruit growth and quality. Plants 13 (14), 1942. doi: 10.3390/plants13141942. PMID: 39065469 PMC11280748

[B20] Barlas N. T. Kadyampakeni D. M. (2022). Citrus responses against nutritional imbalance. In Citrus Production: Technological Advancements and Adaptation to Changing Climate. Eds. Hussain S. Khalid M. F. Ali M. A. Ahmed N. Hasanuzzaman M. Ahmad S. . (Boca Raton, FL, USA: CRC Press), 1–20. doi: 10.1201/9781003119852 (Accessed May 15, 2026).

[B21] Bhatana P. Mousa M. G. Malla R. Khadka D. Shrestha R. K. Vista S. P. (2022). Foliar versus soil biofortification of Zn in citrus (Citrus reticulata Blanco): effect on mineral nutrition, fruit yield, and quality. Biomed. J. Sci. Technol. Res. 41 (5), 076612.

[B22] Bibi F. Rahman A. (2023). An overview of climate change impacts on agriculture and their mitigation strategies. Agriculture 13 (8), 1508. doi: 10.3390/agriculture13081508

[B23] Boaretto R. Hippler F. Ferreira G. Azevedo R. Quaggio J. Mattos D. (2020). The possible role of extra magnesium and nitrogen supply to alleviate stress caused by high irradiation and temperature in lemon trees. Plant Soil 457, 57–70. doi: 10.1007/s11104-020-04597-y

[B24] Boaretto R. Hippler F. Teixeira L. Fornari R. Quaggio J. Mattos D. (2023). Zinc fertilizers for citrus production: assessing nutrient supply via fertigation or foliar application. Plant Soil 596, 1–14. doi: 10.1007/s11104-023-05969-w

[B25] Bonomelli C. Fernández V. Capurro F. Palma C. Videla X. Rojas-Silva X. (2022). Absorption and distribution of calcium (^45^Ca) applied to the surface of orange (Citrus sinensis) fruits at different developmental stages. Agronomy 12 (1), 150. doi: 10.3390/agronomy12010150

[B26] Brasseur G. Stammer D. Friedlingstein P. Hegerl G. Shaw T. Trenberth K. (2025). Climate science for 2050. Front. Clim. 7, 1554685. doi: 10.3389/fclim.2025.1554685

[B27] Brodersen C. Narciso C. Reed M. McElrone A. J. (2014). Phloem production in Huanglongbing-affected citrus trees. HortScience 49 (1), 59–64. doi: 10.21273/HORTSCI.49.1.59

[B28] Cai Y. Zhang H. Qi Y. Ye X. Huang Z. Guo J. (2019). Responses of reactive oxygen species and methylglyoxal metabolisms to magnesium-deficiency differ greatly among the roots, upper and lower leaves of Citrus sinensis. BMC Plant Biol. 19, 455. doi: 10.1186/s12870-019-1683-4. PMID: 30770733 PMC6377732

[B29] Calleja-Cabrera J. Boter M. Oñate-Sánchez L. Pernas M. (2020). Root growth adaptation to climate change in crops. Front. Plant Sci. 11, 544. doi: 10.3389/fpls.2020.00544. PMID: 32457782 PMC7227386

[B30] Cao S. Zhou Y. Zhou Y. Zhou X. Zhou W. (2021). Organic carbon and soil aggregate stability associated with aggregate fractions in a chrono-sequence of citrus orchards plantations. J. Environ. Manage. 293, 112847. doi: 10.1016/j.jenvman.2021.112847 34052614

[B31] Chen H. Chen X. Zheng Z. Huang W. Guo J. Yang L. (2022). Characterization of copper-induced release of exudates by Citrus sinensis roots and their possible roles in copper-tolerance. Chemosphere 308, 111594. doi: 10.1016/j.chemosphere.2022.136348. PMID: 36087738

[B32] Chen H. Hu W. Wang Y. Zhang P. Zhou Y. Yang L. (2023). Declined photosynthetic nitrogen use efficiency under ammonium nutrition is related to photosynthetic electron transport chain disruption in citrus plants. Sci. Hortic. 308, 111594. doi: 10.1016/j.scienta.2022.111594

[B33] da Silva Costa L. Freschi L. Coelho Filho M. A. Araújo da Silva M. A. dos Santos Nascimento F. da Silva Gesteira A. (2025). Reassessing drought tolerance in citrus tetraploid rootstocks: myth or reality? Physiol. Plant. 177 (3), e70199. doi: 10.1111/ppl.70199. PMID: 40171952 PMC11963228

[B34] De Souza A. Cristofani-Yaly M. Da Conceição P. Devite F. Bastianel M. Romero P. (2025). Physiological and productivity responses of Tahiti acid lime grafted onto dwarfing rootstocks under different planting and mulching practices. Front. Sustain. Food Syst. 9, 1489291. doi: 10.3389/fsufs.2025.1489291

[B35] Dong Z. Chen M. Srivastava A. K. Mahmud H. Ishfaq M. Shi X. (2024). Climate changes altered the citrus fruit quality: a 9-year case study in China. Sci. Total Environ. 923, 171406. doi: 10.1016/j.scitotenv.2024.171406 38432361

[B36] Dong Z. Shi X. Liu X. Srivastava A. K. Shi X. Zhang Y. (2025). Calcium application regulates fruit cracking by cross-linking of fruit peel pectin during young fruit growth stage of citrus. Sci. Hortic. 340, 113922. doi: 10.1016/j.scienta.2024.113922

[B37] Dong Z. Srivastava A. K. Liu X. D. Riaz M. Yu Gao Y. Liang X. M. (2021). Interactions between nutrient and Huanglongbing pathogen in citrus: an overview and implications. Sci. Hortic. 290, 110511. doi: 10.1016/j.scienta.2021.110511

[B38] Dovis V. Erismann N. Machado E. Quaggio J. Boaretto R. De Mattos D. (2020). Biomass partitioning and photosynthesis in the quest for nitrogen use efficiency for citrus tree species. Tree Physiol. 41 (3), 349–364. doi: 10.1093/treephys/tpaa126. PMID: 33032323

[B39] Duan J. Liu Y. Yang J. Tang C. Shi Z. (2020). Role of groundcover management in controlling soil erosion under extreme rainfall in citrus orchards of southern China. J. Hydrol. 582, 124290. doi: 10.1016/j.jhydrol.2019.124290

[B40] Fajardo J. De Souza Júnior J. Shahid M. Hammond W. Diepenbrock L. Kadyampakeni D. (2025). Silicon and phosphorus impacts on seasonal nutrient dynamics and tree performance of Citrus sinensis L. under endemic Huanglongbing conditions. Front. Plant Sci. 16, 1651108. doi: 10.3389/fpls.2025.1651108 41200482 PMC12585970

[B41] Fan Z. Xiong H. Luo Y. Wang Y. Zhao H. Li W. (2020). Fruit yields depend on biomass and nutrient accumulations in new shoots of citrus trees. Agronomy 10 (12), 1988. doi: 10.3390/agronomy10121988

[B42] Fan Z. Wu Y. Zhao L. Fu L. Deng L. Deng J. (2022). MYB308-mediated transcriptional activation of plasma membrane H^+^-ATPase promotes iron uptake in citrus. Hortic. Res. 9, uhac088. doi: 10.1093/hr/uhac088. PMID: 35685222 PMC9171118

[B43] Fernando D. Lynch J. (2015). Manganese phytotoxicity: new light on an old problem. Ann. Bot. 116 (3), 313–319. doi: 10.1093/aob/mcv111. PMID: 26311708 PMC4549964

[B44] Ferreira G. Hippler F. Prado L. Rima J. Boaretto R. Quaggio J. (2020). Boron modulates the plasma membrane H^+^-ATPase activity affecting nutrient uptake of citrus trees. Ann. Appl. Biol. 177 (3), 390–401. doi: 10.1111/aab.12630

[B45] Franco-Navarro J. D. Padilla Y. G. Álvarez S. Calatayud Á. Colmenero-Flores J. M. Gómez-Bellot M. J. (2025). Advancements in water-saving strategies and crop adaptation to drought: a comprehensive review. Physiol. Plant. 177 (4), e70332. doi: 10.1111/ppl.70332. PMID: 40599019 PMC12215295

[B46] Fu X.-Z. Xing F. Cao L. Chun C.-P. Ling L.-L. Jiang C.-L. (2016). Effect of foliar application of different zinc fertilizers with organosilicon on correction of citrus zinc deficiency. HortScience 51 (4), 422–426. doi: 10.21273/HORTSCI.51.4.422

[B47] García-Sánchez F. Simón-Grao S. Martínez-Nicolás J. Alfosea-Simón M. Liu C. Chatzissavvidis C. (2020). Multiple stresses occurring with boron toxicity and deficiency in plants. J. Hazard. Mater. 397, 122713. doi: 10.1016/j.jhazmat.2020.122713. PMID: 32402955

[B48] Ghassemi-Golezani K. Farhangi-Abriz S. (2021). Plant responses to exogenous salicylic and jasmonic acids under drought stress. In Jasmonates and Salicylates Signaling in Plants. Eds. Aftab T. Yusuf M. . (Cham, Switzerland: Springer), 65–85. doi: 10.1007/978-3-030-75805-9-4 (Accessed May 15, 2026).

[B49] Giannakoula A. Therios I. Chatzissavvidis C. (2021). Effect of lead and copper on photosynthetic apparatus in Citrus (Citrus aurantium L.) plants. The role of antioxidants in oxidative damage as a response to heavy metal stress. Plants 10 (1), 155. doi: 10.3390/plants10010155. PMID: 33466929 PMC7830311

[B50] Gojon A. Cassan O. Bach L. Lejay L. Martin A. (2022). The decline of plant mineral nutrition under rising CO_2_: physiological and molecular aspects of a bad deal. Trends Plant Sci. 28 (2), 185–198. doi: 10.1016/j.tplants.2022.09.002. PMID: 36336557

[B51] Gong K. Wang N. Chen Y. Yu J. Kuang C. Xiong X. (2026). Enhancing iron nutrition in citrus: synergising roles of proline-2'-deoxymuginineic acid in root physiology and microbiome. J. Agric. Food Chem. 74, 1998–2011. doi: 10.1021/acs.jafc.5c09250 41510663

[B52] Graham J. H. Johnson E. G. Gottwald T. R. Irey M. S. (2013). Presymptomatic fibrous root decline in citrus trees caused by huanglongbing and potential interaction with water deficit. Plant Dis. 97 (9), 1195–1199. doi: 10.1094/PDIS-01-13-0024-RE 30722426

[B53] Guo L. Li Y. Yu Z. Wu J. Jin J. Liu X. (2021). Interactive influences of elevated atmospheric CO_2_ and temperature on phosphorus acquisition of crops and its availability in soil: a review. Int. J. Plant Prod. 15 (2), 173–182. doi: 10.1007/s42106-021-00138-4

[B54] Guo H. Zheng Y. Wu D. Du X. Gao H. Ayyash M. (2023). Quality evaluation of citrus varieties based on phytochemical profiles and nutritional properties. Front. Nutr. 10, 1165841. doi: 10.3389/fnut.2023.1165841. PMID: 37275647 PMC10232803

[B55] Gupta S. Bantawa P. Rai S. Ali S. Pradhan J. Lama S. (2024). Enhancing citrus resilience: strategies and advances in abiotic stress management: a review. Indian J. Agric. Res. 58 (special issue), 953–961. doi: 10.18805/ijare.A-6270

[B56] Hamido S. A. Ebel R. C. Morgan K. (2019). Interaction of huanglongbing and foliar applications of copper on water relations of Citrus sinensis cv. Valencia. Plants 8 (9), 298. doi: 10.3390/plants8090298. PMID: 31443580 PMC6784184

[B57] Hauer-Jákli M. Tränkner M. (2019). Critical leaf magnesium thresholds and the impact of magnesium on plant growth and photo-oxidative defense: a systematic review and meta-analysis from 70 years of research. Front. Plant Sci. 10, 766. doi: 10.3389/fpls.2019.00766. PMID: 31275333 PMC6592071

[B58] Hippler F. W. R. Boaretto R. M. Dovis V. L. Quaggio J. A. Azevedo R. A. Mattos-Junior D. (2018). Oxidative stress induced by Cu nutritional disorders in citrus depends on nitrogen and calcium availability. Sci. Rep. 8, 1641. doi: 10.1038/s41598-018-19735-x. PMID: 29374264 PMC5786063

[B59] Hippler F. W. R. Boaretto R. M. Quaggio J. A. Boaretto A. E. Abreu-Junior C. H. Mattos Junior D. (2015). Uptake and distribution of soil applied zinc by citrus trees—addressing fertilizer use efficiency with ^68^Zn labeling. PLoS One 10 (4), e0116903. doi: 10.1371/journal.pone.0116903. PMID: 25751056 PMC4353711

[B1300] Hu X. Wang J. Wu F. Li D. Yang J. Chen J. . (2023). Phosphorus recovery and resource utilization from phosphogypsum leachate via membrane-triggered adsorption and struvite crystallization approach. Chem. Engg. J. 471, 144310. doi: 10.1016/j.cej.2023.144310

[B62] Huan H. Hu C.-X. Tan Q. Hu X. Sun K. Bi L. (2012). Effect of Fe-EDDHA application on iron chlorosis of citrus trees and comparison of evaluations on nutrient balance with three approaches. Sci. Hortic. 146, 137–142. doi: 10.1016/j.scienta.2012.08.015

[B61] Huang W. Chen X. Huang W. Shen Q. Lu F. Lai N. (2025). Humic acid enhances antioxidant and glyoxalase systems to combat copper toxicity in citrus. Agronomy 15 (1), 99. doi: 10.3390/agronomy15010099

[B63] Huang W. Xie Y. Chen X. Zhang J. Chen H. Ye X. (2021). Growth, mineral nutrients, photosynthesis and related physiological parameters of citrus in response to nitrogen deficiency. Agronomy 11 (9), 1859. doi: 10.3390/agronomy11091859

[B64] Huang W. Zheng Z. Hua D. Chen X. Zhang J. Chen H. (2022). Adaptive responses of carbon and nitrogen metabolisms to nitrogen-deficiency in Citrus sinensis seedlings. BMC Plant Biol. 22, 387. doi: 10.1186/s12870-022-03759-7. PMID: 35879653 PMC9316421

[B65] Hussain S. Karagiannis E. Manzoor M. Ziogas V. (2023). From stress to success: harnessing technological advancements to overcome climate change impacts in citriculture. Crit. Rev. Plant Sci. 42 (5), 345–363. doi: 10.1080/07352689.2023.2248438

[B66] Ishfaq M. Wang Y. Yan M. Wang Z. Wu L. Li C. (2022). Physiological essence of magnesium in plants and its widespread deficiency in the farming system of China. Front. Plant Sci. 13, 802274. doi: 10.3389/fpls.2022.802274. PMID: 35548291 PMC9085447

[B67] James H. Graham R. B. Bassanezi W. O. Dawson R. D. (2024). Management of huanglongbing of citrus: lessons from São Paulo and Florida. Annu. Rev. Phytopathol. 62, 243–262. doi: 10.1146/annurev-phyto-121423-041921. PMID: 38691871

[B68] Jiao Y. Shao C. Shu Q. (2022). Integrated physiological and metabolomic analyses of the effect of potassium fertilizer on citrus fruit splitting. Plants 11 (4), 499. doi: 10.3390/plants11040499. PMID: 35214832 PMC8877888

[B69] Johnson E. G. Wu J. Bright B. Graham J. H. (2014). Association of 'Candidatus Liberibacter asiaticus' root infection, but not phloem plugging with root loss on huanglongbing-affected trees prior to appearance of foliar symptoms. Plant Pathol. 63 (2), 290–298. doi: 10.1111/ppa.12109

[B70] Kadyampakeni D. M. Morgan K. T. Schumann A. W. Nkedi-Kizza P. (2014). Effect of irrigation pattern and timing on root density of young citrus trees infected with huanglongbing disease. HortTechnology 24 (2), 209–221. doi: 10.21273/HORTTECH.24.2.209

[B71] Kadyampakeni D. Nkedi-Kizza P. Leiva J. Muwamba A. Fletcher E. Morgan K. (2018). Ammonium and nitrate transport during saturated and unsaturated water flow through sandy soils. J. Plant Nutr. Soil Sci. 181 (2), 198–210. doi: 10.1002/jpln.201700405

[B72] Karamidehkordi E. Sadati S. Tajvar Y. Mirmousavi S. (2023). Climate change vulnerability and resilience strategies for citrus farmers. Environ. Sustain. Indic. 20, 100317. doi: 10.1016/j.indic.2023.100317

[B74] Keteku A. K. Yeboah P. A. Yeboah S. Dormatey R. Agyeman K. Brempong M. B. (2024). Plant nutrition in relation to water-use efficiency in crop production: a review. J. Sustain. Agric. 17 (1), 67–95. doi: 10.37478/agr.v17i1.3547

[B75] Khan H. Chunjie Q. (2026). Predicting the future of citrus: country-level forecasts for production in leading producer nations. Appl. Fruit Sci. 68, 91. doi: 10.1007/s10341-026-01800-9

[B76] Khan M. N. Hayat F. Asim M. Iqbal S. Ashraf T. Asghar S. (2020). Influence of citrus rootstocks on growth performance and leaf mineral nutrition of 'Salustiana' sweet orange [Citrus sinensis (L.) Obsek]. J. Pure Appl. Agric. 5 (2), 46–53.

[B77] Kwakye S. Kadyampakeni D. M. (2022). Micronutrients improve growth and development of HLB-affected citrus trees in Florida. Plants 12 (1), 73. doi: 10.3390/plants12010073. PMID: 36616206 PMC9824839

[B78] Kwakye S. Kadyampakeni D. M. (2023). Impact of deficit irrigation on growth and water relations of HLB-affected citrus trees under greenhouse conditions. Water 15 (11), 2085. doi: 10.3390/w15112085

[B79] Kwakye S. Kadyampakeni D. Vashisth T. (2020). Influence of elevated manganese rates on growth parameters, nutrient, and biomass accumulation of HLB-affected trees in Florida. Proc. Fla. State Hortic. Soc. 133, 42–46.

[B80] Lado J. Gambetta G. Zacarías L. (2018). Key determinants of citrus fruit quality: metabolites and main changes during maturation. Sci. Hortic. 233, 238–248. doi: 10.1016/j.scienta.2018.01.055

[B81] Lea-Cox J. Syvertsen J. Graetz D. (2001). Springtime ^15^nitrogen uptake, partitioning, and leaching losses from young bearing citrus trees of differing nitrogen status. J. Am. Soc. Hortic. Sci. 126 (2), 242–251. doi: 10.21273/JASHS.126.2.242

[B82] Li S. Long Y. Deng G. Men Y. Lu F. Wang Z. (2025). Manganese-based nanozyme enabled efficient mitigation of huanglongbing-induced oxidative damage in citrus. Environ. Sci. Nano 12, 701–715. doi: 10.1039/D4EN00519H

[B83] Lidon A. Ramos C. Ginestar D. Contreras W. (2013). Assessment of LEACHN and a simple compartmental model to simulate nitrogen dynamics in citrus orchards. Agric. Water Manag. 121, 42–53. doi: 10.1016/j.agwat.2013.01.008

[B84] Lie Z. Zhou G. Huang W. Kadowaki K. Tissue D. Yan J. (2021). Warming drives sustained plant phosphorus demand in a humid tropical forest. Global Change Biol. 28 (13), 4085–4096. doi: 10.1111/gcb.16194. PMID: 35412664

[B85] Liu X. Hu C. Liu X. Riaz M. Liu Y. Dong Z. (2022). Effect of magnesium application on the fruit coloration and sugar accumulation of navel orange (Citrus sinensis Osb.). Sci. Hortic. 304, 111282. doi: 10.1016/j.scienta.2022.111282

[B86] Lu X. Zhao C. Shi H. Liao Y. Xu F. Du H. (2021). Nutrients and bioactives in citrus fruits: different citrus varieties, fruit parts, and growth stages. Crit. Rev. Food Sci. Nutr. 63 (13), 2018–2041. doi: 10.1080/10408398.2021.1969891. PMID: 34609268

[B87] Luo Z. Zhang L. Hu W. Wang Y. Tao J. Jia Y. (2024). Excessive boron fertilization-induced toxicity is related to boron transport in field-grown pomelo trees. Front. Plant Sci. 15, 1438664. doi: 10.3389/fpls.2024.1438664. PMID: 39319002 PMC11420558

[B88] Ma W. Pang Z. Huang X. Xu J. Pandey S. S. Li J. (2022). Citrus huanglongbing is an immune-mediated disease that can be mitigated by antioxidants and gibberellin. Nat. Commun. 13, 529. doi: 10.1038/s41467-022-28122-6 35082290 PMC8791970

[B107] Maharajan T. Caesar S. A. Palayullaparambil A. K. Ignacimuthu S. (2021a). Management of phosphorous nutrient and climate change for sustainable agriculture. J. Environ. Qual. 50 (6), 1303–1324. doi: 10.1002/jeq2.20292. PMID: 34559407

[B90] Martínez-Cuenca M. Martínez-Alcántara B. Millos J. Legaz F. Quiñones A. (2021). Seasonal Fe uptake of young citrus trees and its contribution to the development of new organs. Plants 10 (1), 79. doi: 10.3390/plants10010079. PMID: 33401714 PMC7823581

[B91] Martínez-Cuenca M. Quiñones A. Primo-Millo E. Forner-Giner M. (2015). Flooding impairs Fe uptake and distribution in citrus due to the strong down-regulation of genes involved in strategy I responses to Fe deficiency in roots. PLoS One 10 (4), e0123644. doi: 10.1371/journal.pone.0123644. PMID: 25897804 PMC4405480

[B92] Mattos Junior D. Hippler F. W. R. Boaretto R. M. Stuchi E. S. Quaggio J. A. (2017). Soil boron fertilization: the role of nutrient sources and rootstocks in citrus production. J. Integr. Agric. 16 (7), 1609–1616. doi: 10.1016/S2095-3119(16)61492-2

[B93] Mattos Junior D. Ramos U. M. Quaggio J. A. Furlani P. R. (2010). Nitrogen and copper for citrus nursery production on two different rootstocks. Bragantia 69 (1), 125–132. doi: 10.1590/S0006-87052010000100018

[B94] Medda S. Fadda A. Mulas M. (2022). Influence of climate change on metabolism and biological characteristics in perennial woody fruit crops in the Mediterranean environment. Horticulturae 8 (4), 273. doi: 10.3390/horticulturae8040273

[B95] Meng X. Chen W. Wang Y. Huang Z. Ye X. Chen L. (2021). Effects of phosphorus deficiency on the absorption of mineral nutrients, photosynthetic system performance and antioxidant metabolism in Citrus grandis. PLoS One 16 (2), e0246944. doi: 10.1371/journal.pone.0246944. PMID: 33596244 PMC7888624

[B96] Mesejo C. Martínez-Fuentes A. Reige C. El-Otmani M. Agustí M. (2024). Examining the impact of dry climates temperature on citrus fruit internal ripening. Sci. Hortic. 337, 113501. doi: 10.1016/j.scienta.2024.113501

[B97] Meshram D. Srivastava A. K. Utkhede A. Pangul C. Ziogas V. (2025). Response to sensor-based fertigation of Nagpur mandarin (Citrus reticulata Blanco) in Vertisol of Central India. Horticulturae 11 (5), 508. doi: 10.3390/horticulturae11050508

[B98] Mesquita G. L. Zambrosi F. C. B. Tanaka F. A. O. Boaretto R. M. Quaggio J. A. Ribeiro R. V. (2016). Anatomical and physiological responses of citrus trees to varying boron availability are dependent on rootstock. Front. Plant Sci. 7, 224. doi: 10.3389/fpls.2016.00224 26973670 PMC4777737

[B99] Mishra S. Spaccarotella K. Gido J. Samanta I. Chowdhary G. (2023). Effects of heat stress on plant-nutrient relations: an update on nutrient uptake, transport, and assimilation. Int. J. Mol. Sci. 24 (21), 15670. doi: 10.3390/ijms242115670. PMID: 37958654 PMC10649217

[B100] Mo X. Chen C. Riaz M. Moussa M. Chen X. Wu S. (2022). Fruit characteristics of citrus trees grown under different soil Cu levels. Plants 11 (21), 2943. doi: 10.3390/plants11212943. PMID: 36365397 PMC9657546

[B101] Modica G. Arcidiacono F. Puglisi I. Baglieri A. La Malfa S. Gentile A. (2025). Response to water stress of eight novel and widely spread citrus rootstocks. Plants 14 (5), 773. doi: 10.3390/plants14050773. PMID: 40094758 PMC11901693

[B102] Morade A. S. Sharma R. M. Dubey A. K. Sathee L. Kumar S. Kadam D. M. (2025). Phenotyping drought stress tolerance in citrus rootstocks using high-throughput imaging and physio-biochemical techniques. BMC Plant Biol. 25, 753. doi: 10.1186/s12870-025-06823-0. PMID: 40468193 PMC12135332

[B103] Morales J. Martínez-Alcántara B. Bermejo A. Millos J. Legaz F. Quiñones A. (2023). Effect of calcium fertilization on calcium uptake and its partitioning in citrus trees. Agronomy 13 (12), 2971. doi: 10.3390/agronomy13122971

[B104] Moreno-Lora A. Calero-Velázquez R. Arenas-Arenas F. (2025). Physiological responses of new citrus rootstocks to salinity stress induced by increasing sodium and chloride concentrations in the irrigation solution. J. Soil Sci. Plant Nutr. 25, 7868–7877. doi: 10.1007/s42729-025-02637-z

[B105] Morgan K. T. Graham J. H. (2019). Nutrient status and root density of Huanglongbing-affected trees: consequences of irrigation water bicarbonate and soil pH mitigation with acidification. Agronomy 9 (11), 746. doi: 10.3390/agronomy9110746

[B106] Morgan K. T. Rouse R. E. Ebel R. C. (2016). Foliar applications of essential nutrients on growth and yield of 'Valencia' sweet orange infected with Huanglongbing. HortScience 51 (12), 1482–1493. doi: 10.21273/HORTSCI11026-16

[B108] Mousavi S. M. Srivastava A. K. Raiesi T. (2024). Citrus nutrition Iran: lessons from calcareous soils. J. Plant Nutr. 47 (19), 1–26. doi: 10.1080/01904167.2024.2379571

[B109] Mushtaq M. Mushtaq M. Ateeq M. Alan S. Ashraf M. Kashif H. (2024). Combating smog in Pakistan's Kinnow industry: nanotechnology and sustainable resource management for resilient agriculture. J. Hortic. Sci. Technol. 7 (3), 73–79. doi: 10.46653/jhst24073073

[B110] Naeem S. Sami A. Haider M. Ali M. Khaliq A. Akram M. (2024). Heat stress in citrus: a molecular functional and biochemical perception. Bull. Biol. Allied Sci. Res. 2024 (1), 69. doi: 10.54112/bbasr.v2024i1.69

[B111] Nasiro K. Mohammednur T. (2024). Precision nutrient management amid climate change challenges: a review. Front. Plant Sci. 5 (3), 110–122. doi: 10.11648/j.sf.20240503.12

[B112] Navarro J. Antolinos V. Robles J. Botía P. (2022). Citrus irrigation with desalinated seawater under a climate change scenario. Front. Plant Sci. 13, 909083. doi: 10.3389/fpls.2022.909083. PMID: 35707618 PMC9190299

[B113] Navarro-Gochicoa M. Aparicio-Durán L. Delfín A. Ceacero C. Herrera-Rodriguez M. Arenas-Arenas F. (2024). Characterization of citrus rootstock under conditions of boron toxicity. Agronomy 14 (11), 2741. doi: 10.3390/agronomy14112741

[B114] Navarro-Pérez V. Simón I. Cámara-Zapata J. Muñoz-Acero J. Alfosea-Simón M. García-Sánchez F. (2024). Effects of high boron concentration in irrigation water on the relative tolerance and metabolic responses of different citrus varieties: lemon, orange, and mandarin. Sci. Hortic. 338, 113660. doi: 10.1016/j.scienta.2024.113660

[B115] Nawaz R. Abbasi N. Hafiz I. Khan M. Khalid A. (2021). Environmental variables influence the developmental stages of the citrus leaf miner, infestation level and mined leaves physiological response of Kinnow mandarin. Sci. Rep. 11, 864. doi: 10.1038/s41598-021-87160-8. PMID: 33833311 PMC8032831

[B116] Nicolás-Espinosa J. Yepes-Molina L. Martínez-Bernal F. Fernández-Pozurama M. Carvajal M. (2024). Deciphering the effect of salinity and boron stress on broccoli plants reveals that membrane phytosterols and PIP aquaporins facilitate stress adaptation. Plant Sci. 338, 111923. doi: 10.1016/j.plantsci.2023.111923 37972760

[B117] Nieves-Cordones M. Ródenas R. Lara A. Martínez V. Rubio F. (2018). The combination of K^+^ deficiency with other environmental stresses: what is the outcome? Physiol. Plant. 165 (2), 264–276. doi: 10.1111/ppl.12827. PMID: 30187486

[B118] Ortiz-Bobea A. Ault T. Carrillo C. Chambers R. Lobell D. (2021). Anthropogenic climate change has slowed global agricultural productivity growth. Nat. Clim. Change 11, 306–312. doi: 10.1038/s41558-021-01000-1

[B119] Osorio-Marín J. Fernández E. Vieli L. Ribera A. Luedeling E. Cobo N. (2024). Climate change impacts on temperate fruit and nut production: a systematic review. Front. Plant Sci. 15, 1352169. doi: 10.3389/fpls.2024.1352169. PMID: 38567135 PMC10986187

[B1200] Pang F. Li Q. Solanki M. K. Wang Z. Xing Y-X. Dong D-F. (2024). Soil phosphorus transformation and plant uptake driven by phosphate-solubilizing microorganisms. Front. Microbiol. 15, 1383813. doi: 10.3389/fmicb.2024.1383813 38601943 PMC11005474

[B121] Panigrahi P. Srivastava A. K. Panda D. K. Huchche A. D. (2017). Rainwater, soil and nutrients conservation for improving productivity of citrus orchards in a drought prone region. Agric. Water Manage. 185, 65–77. doi: 10.1016/j.agwat.2017.02.009

[B122] Papadakis I. Ladikou E. Oikonomou A. Chatzistathis T. Chatziperou G. (2023b). Exploring the impact of potassium on growth, photosynthetic performance, and nutritional status of lemon trees (cv. Adamopoulou) grafted onto sour orange and Volkamer lemon rootstocks. Sustainability 15 (22), 15858. doi: 10.3390/su152215858

[B123] Papadakis I. E. Satirpoulos T. E. Therios I. N. (2006). Mobility of iron and manganese within two citrus genotypes after foliar applications of iron sulphate and manganese sulphate. J. Plant Nutr. 30 (9), 1385–1396. doi: 10.1080/01904160701557540

[B1400] Pereira S. Monteiro A. Baltazar M. Maia C. Pereira S. Oliveira M. J. . (2025). Modulating grapevine performance and hormonal dynamics under summer stress by the synergistic effects of kaolin and silicon. Front. Plant Sci. 16, 1639169. doi: 10.3389/fpls.2025.1639169 40842520 PMC12365603

[B124] Pestana M. García-Caparrós P. Saavedra T. Gama F. Abadía J. Varennes A. (2023). Nutritional performance of five citrus rootstocks under different Fe levels. Plants 12 (18), 3252. doi: 10.3390/plants12183252. PMID: 37765416 PMC10535202

[B125] Pestana M. Varennes A. Abadia J. Faria E. A. (2005). Differential tolerance to iron deficiency of citrus rootstocks grown in nutrient solution. Sci. Hortic. 104 (1), 25–36. doi: 10.1016/j.scienta.2004.07.007

[B126] Pilon L. C. Ambus J. V. Blume E. Jacques R. J. S. Reichert J. M. (2023). Citrus orchards in agroforestry, organic, and conventional systems: soil quality and functioning sustainability. Sustainability 15 (17), 13060. doi: 10.3390/su151713060

[B127] Putri I. H. S. Handayani W. Mulia F. A. Ichihara J. Anggraeni M. Setiadi R. (2025). Examination of barriers to climate change adaptations-experience from Semarang city, Indonesia. Urban Gov. 5 (3), 314–330. doi: 10.1016/j.ugj.2025.08.005

[B128] Qaderi M. Evans C. Spicer M. (2025). Plant nitrogen assimilation: a climate change perspective. Plants 14 (7), 1025. doi: 10.3390/plants14071025. PMID: 40219093 PMC11990535

[B129] Qin W. Assinck F. T. B. Heinenb M. Oenema O. (2016). Water and nitrogen use efficiencies in citrus production: a meta-analysis. Agric. Ecosyst. Environ. 222, 101–111. doi: 10.1016/j.agee.2016.01.052

[B130] Qu M. Huang X. Garcia-Caparros P. Shabala L. Fuglsang A. T. Yu M. (2024). Understanding the role of boron in plant adaptation to salinity. Physiol. Plant. 176 (3), e14358. doi: 10.1111/ppl.14358. PMID: 38783511

[B131] Quaggio J. Souza T. Zambrosi F. Mattos Junior D. Boaretto R. Silva G. (2019). Citrus fruit yield response to nitrogen and potassium fertilization depends on nutrient-water management system. Sci. Hortic. 252, 315–322. doi: 10.1016/j.scienta.2019.02.001

[B132] Rao R. Huang W. Yang H. Shen Q. Huang W. Lu F. (2025). Raising pH reduces manganese toxicity in Citrus grandis (L.) Osbeck by efficient maintenance of nutrient homeostasis to enhance photosynthesis and growth. Plants 14 (15), 2390. doi: 10.3390/plants14152390 40805740 PMC12349334

[B444] Rao R.-Y. Lan B.-B. Zhu X. Huang W. L. Chen X. -F. Lai N.-W. . (2026). Augmenting pH confers Citrus grandis the ability to combat oxidative stress triggered by manganese excess. Plants (Basel). 151, 172. doi: 10.3390/plants15010172 PMC12787632 41515116

[B133] Rasera J. Da Silva R. Filho F. Delbem A. Saraiva A. Sentelhas P. (2023). Climate change and citriculture: a bibliometric analysis. Agronomy 13 (3), 723. doi: 10.3390/agronomy13030723

[B134] Razi M. F-U-D. Khan I. A. Jaskani M. J. (2011). Citrus plant nutritional profile in relation to huanglongbing prevalence in Pakistan. Pak. J. Agric. Sci. 48 (4), 297–304.

[B135] Rehman S. Shafqat W. Ikram S. Chattha W. Amen R. Deng H. (2024). Citrus rootstocks physiological and anatomical response to heat stress. Acta Physiol. Plant. 46, 176. doi: 10.1007/s11738-024-03709-w

[B136] Rivera-Hernández B. Garruña R. Andrade J. L. Tezara W. Us Santamaría R. Andueza-Noh R. H. (2025). Seasonal changes in physiological responses and yield of Citrus latifolia under high-density planting and different soil moisture tensions. Horticulturae 11 (12), 1472. doi: 10.3390/horticulturae11121472

[B137] Rodríguez-Azorín L. Gómez-Cádenas A. López-Climent M. Vives-Peris V. (2025). Integrated analysis of multifactorial stress combination impact on citrus plants. Planta 262, 80. doi: 10.1007/s00425-025-04866-z. PMID: 41191095 PMC12589267

[B138] Rymbai H. Parent L.-E. Srivastava A. K. Talang H. Verma V. K. Ziogas V. (2026). Machine learning tools-based diagnosis of soil nutrient constraints to increase the productivity of citrus orchards. Front. Agron. 8, 1719690. doi: 10.3389/fagro.2026.1719690

[B139] Segovia C. Gomez J. D. Gallardo P. Lozano F. J. Asensio C. (2017). Soil nutrients losses by wind erosion in citrus crop at southeast Spain. Eurasian Soil Sci. 50 (6), 756–763. doi: 10.1134/S1064229317060114

[B140] Shafaqat W. Naqvi S. A. Maqbool R. Haider M. S. Jaskani M. J. Khan J. A. (2021). Climate change and citrus. IntechOpen. (London, UK: IntechOpen). doi: 10.5772/intechopen.95488 (Accessed May 15, 2026).

[B141] Shahzad F. Chun C. Schumann A. Vashisth T. (2020). Nutrient uptake in Huanglongbing-affected sweet orange: transcriptomic and physiological analysis. J. Am. Soc. Hortic. Sci. 145 (5), 349–362. doi: 10.21273/JASHS04929-20

[B142] Shahzad F. Vashisth T. Ritenour M. A. Wang Y. Brecht J. K. (2025). Supplemental nutrition mitigates Huanglongbing symptoms, and improves fruit quality and shelf-life of 'LB8-9' (Sugar Belle) and 'Tango' mandarins. J. Am. Soc. Hortic. Sci. 150 (5), 213–225. doi: 10.21273/JASHS05500-25

[B143] Sheng X. Li M. Luo Y. Mao Z. Zhai X. Liu J.-H. (2025). The CsMyB36-CsSWEET17 module mediates calcium-induced sucrose accumulation in citrus. Hortic. Res. 12, uhaf175. doi: 10.1093/hr/uhaf175 41112969 PMC12528650

[B144] Shirgure P. S. Srivastava A. K. Singh S. (2001). Nitrogen fertigation and band placement fertilizer application in soil-leaf buildup and incremental growth in acid lime. J. Soil Water Conserv. 45 (3), 176–181.

[B145] Simon-Grao S. Nieves M. Martinez-Nicolas J. J. Camara-Zapata J. M. Alfosea-Simon M. Garcia-Sanchez F. (2018). Response of three citrus genotypes used as rootstocks grown under boron excess conditions. Ecotoxicol. Environ. Saf. 159, 10–15. doi: 10.1016/j.ecoenv.2018.04.042 29730402

[B146] Soares J. Santos C. Carvalho S. Pintado M. Vasconcelos M. (2019). Preserving the nutritional quality of crop plants under a changing climate: importance and strategies. Plant Soil 443, 1–26. doi: 10.1007/s11104-019-04229-0

[B147] Soliman S. Rezk A. Rocha F. Rodriguez-Ramos J. Manoharan B. Wang Y. (2025). Strigolactone GR24 modulates citrus root architecture and rhizosphere microbiome under nitrogen and phosphorus deficiency. BMC Plant Biol. 25, 698. doi: 10.1186/s12870-025-07515-5. PMID: 41239215 PMC12619333

[B148] Srivastava A. K. (2024). Challenges of plant nutrition and climate change: focus on fruit crops. In Silicon Advances for Sustainable Agriculture and Human Health: Increased Nutrition and Disease Prevention. (Cham, Switzerland: Springer Nature), 1–40. doi: 10.1007/978-3-031-69876-7_1 (Accessed July 10, 2024).

[B149] Srivastava A. K. Das A. K. Jagannadham P. K. Bora P. Ansari F. A. Bhate R. (2022). Bioprospecting soil and plant health management amidst Huanglongbing threat in citrus; a review. Front. Plant Sci. 13, 858842. doi: 10.3389/fpls.2022.858842. PMID: 35557712 PMC9088001

[B150] Srivastava A. K. Hota D. (2020). Fruit crops under nutrient-capped scenario: a timeless journey. Curr. Sci. 119 (1), 14–17. doi: 10.18520/cs/v119/i1/14-17

[B151] Srivastava A. K. Majid S. M. Bora P. Hota D. Malhotra S. K. Pandey V. (2025). Rhizosphere to rhizosphere hybridization: new initiatives in fruit crops. Front. Hortic. 4, 1584807. doi: 10.3389/fhort.2025.1584807

[B152] Srivastava A. K. Patil P. (2016). Nutrient indexing in Kinnow mandarin (Citrus delicosa Lour x Citrus nobilis Tanaka) grown in Indo-Gangetic plains. Commun. Soil Sci. Plant Anal. 47 (18), 2115–2125. doi: 10.1080/00103624.2016.1228947

[B153] Srivastava A. K. Singh S. (2004). Zinc nutrition and citrus decline – a review. Agric. Rev. 25 (3), 173–188.

[B154] Srivastava A. K. Singh S. (2005a). Diagnosis of nutrient constraints in citrus orchards of humid tropical India. J. Plant Nutr. 29 (6), 1061–1076. doi: 10.1080/01904160600689183

[B155] Srivastava A. K. Singh S. (2005b). Zinc nutrition, a global concern for sustainable citrus production. J. Sustain. Agric. 25 (3), 42–45. doi: 10.1300/J064v25n03_05

[B156] Stagno F. Randazzo A. Roccuzzo G. Ciorba R. Amoriello T. Ciccoritti R. (2025). Assessment of citrus water status using proximal sensing: a comparative study of spectral and thermal techniques. Land 14 (6), 1222. doi: 10.3390/land14061222

[B157] Sutton M. Vashisth T. (2025). Frequent irrigation increases yield in Huanglongbing-affected sweet orange. HortTechnology 35 (6), 871–879. doi: 10.21273/HORTTECH05733-25

[B158] Tan X. P. Li C. Z. Zou Y. N. Lei A. Q. Alqahtani M. D. Wu Q.-S. (2026). Cultivar-dependent effects of arbuscular mycorrhizal (AM) fungal inoculation on fruit quality and native AM fungal community in navel orange. Rhizosphere 37, 101269. doi: 10.1016/j.rhisph.2026.101269

[B1000] Ter’an F. Vives-Peris V. L´opez-Climent M. F. G’omez-Cadenas A. P´erez-Clemente R. M. (2023). Palliative effects of kaolin on citrus plants under controlled stress conditions of high temperature and high light intensity. J. Plant Growth Regul. 43, 486–499. doi: 10.1007/s00344-023-11103-y

[B159] Thor K. (2019). Calcium-nutrient and messenger. Front. Plant Sci. 10, 440. doi: 10.3389/fpls.2019.00940. PMID: 31073302 PMC6495005

[B160] Tian Y. Shi C. Malo C. Kengdo S. Heinzle J. Inselsbacher E. (2023). Long-term soil warming decreases microbial phosphorus utilization by increasing abiotic phosphorus sorption and phosphorus losses. Nat. Commun. 14, 864. doi: 10.1038/s41467-023-36527-8. PMID: 36792624 PMC9932148

[B161] Torrisi B. Trinchera A. Roccuzzo G. Allegla M. Sequi P. Epifani R. (2015). A new approach in citrus iron chlorosis: organo-mineral fertilizers from glass matrix and organic biofertilizers. Acta Hortic. 1065, 1503–1510. doi: 10.17660/ActaHortic.2015.1065.222

[B162] Tripathi R. Tewari R. Singh K. P. Keswani C. Minkina T. Srivastava A. K. (2022). Plant mineral nutrition and disease resistance: a significant linkage for sustainable crop protection. Front. Plant Sci. 13, 883970. doi: 10.3389/fpls.2022.883970. PMID: 36340341 PMC9631425

[B163] Tutmez B. Dag A. Erden H. Torun B. (2009). Evaluation of Mn concentration provided by soil in citrus-growing regions. Comput. Electron. Agric. 67 (1-2), 27–34. doi: 10.1016/j.compag.2009.02.005

[B164] Uthman Q. Kadyampakeni D. Nkedi-Kizza P. Kwakye S. Barlas N. (2022). Boron, manganese, and zinc sorption and leaf uptake on citrus cultivated on a sandy soil. Plants 11 (5), 638. doi: 10.3390/plants11050638. PMID: 35270107 PMC8912630

[B165] Vashisth T. (2020). Nutrition: No One Size Fits All. (University of Florida, USA: Citrus Industry Magazine). Available online at: https://citrusindustry.net/2020/03/05/nutrition-no-one-size-fits-all/ (Accessed January 10, 2020).

[B166] Vincent C. Morillon R. Arbona V. Gómez-Cadenas A. (2020). Citrus in changing environments. In The Genus Citrus. Eds. Talon M. Caruso M. Gmitter F. G. . (Duxford, UK: Woodhead Publishing), 271–289. doi: 10.1016/B978-0-12-812163-4.00013-9 (Accessed May 15, 2026).

[B167] Vives Peris V. Vicente Valls A. Pérez-Tornero O. (2025). Physiological and biochemical adaptive strategies in citrus plants facing cold stress. Plant Growth Regul. 105, 1575–1590. doi: 10.1007/s10725-025-01361-z

[B168] Wagner Y. Brumfeld V. Grünzweig J. Klein T. (2020). The effect of soil potassium and carbohydrates on xylem conductivity and embolism in an evergreen angiosperm tree and a gymnosperm tree before and after drought. bioRxiv. doi: 10.1101/2020.11.11.379156 (Accessed May 15, 2026).

[B169] Wan Y. X. Lu Y. Rong Z. Y. Zou Y. N. Wu Q.-S. (2025). Host genotype shapes fungal symbiont-mediated nutrient and growth benefits in citrus. Horticulturae 11 (11), 1321. doi: 10.3390/horticulturae11111321

[B170] Wang Y. Long Q. Li Y. Kang F. Fan Z. Xiong H. (2022). Mitigating magnesium deficiency for sustainable citrus production: a case study in Southwest China. Sci. Hortic. 295, 110832. doi: 10.1016/j.scienta.2021.110832

[B171] Wang T. Tan L. Chen Z. Yang Y. Yuan Y. Zheng Z. (2024). Mitigating citrus fruit cracking: the efficacy of chelated calcium or silicon foliar fertilizers in 'Okitsu no. 58' citrus fruit. Front. Plant Sci. 15, 1402945. doi: 10.3389/fpls.2024.1402945. PMID: 39114472 PMC11303202

[B172] Wang S. Wei X. Hao M. (2016). Dynamics and availability of different pools of manganese in semiarid soils as affected by cropping system and fertilization. Pedosphere 26 (3), 351–361. doi: 10.1016/S1002-0160(15)60048-0

[B173] Wei Q. Wei Q. Xu J. Liu Y. Wang D. Chen S. (2024). Nitrogen losses from soil as affected by water and fertilizer management under drip irrigation: development, hotspots and future perspectives. Agric. Water Manag. 296, 108791. doi: 10.1016/j.agwat.2024.108791

[B176] Wu F. Huang H. Peng M. Lai Y. Ren Q. Zhang J. (2021). Adaptive responses of Citrus grandis leaves to copper toxicity revealed by RNA-Seq and physiology. Int. J. Mol. Sci. 22 (21), 12023. doi: 10.3390/ijms222112023. PMID: 34769452 PMC8585100

[B175] Wu K. Hu C. Liao P. Hu Y. Sun X. Tan Q. (2024b). Potassium stimulates fruit sugar accumulation by increasing carbon flow in Citrus sinensis. Hortic. Res. 11, uhae240. doi: 10.1093/hr/uhae240. PMID: 39512779 PMC11540757

[B178] Wu Q.-S. Srivastava A. K. Zou Y. N. Malhotra S. K. (2017). Mycorrhizas in citrus: beyond soil fertility and plant nutrition. Indian J. Agric. Sci. 87 (4), 427–443. doi: 10.56093/ijas.v87i4.69308

[B174] Wu S. Gao G. Du Y. Mo X. Tan Q. Sun X. (2024a). Low soil pH enhances fruit acidity by inhibiting citric acid degradation in lemon (Citrus lemon L.). Hortic. Adv. 2, 44. doi: 10.1007/s44281-024-00044-5

[B177] Wu S. Liang S. Hu C. Tan Q. Zhang J. Dong Z. (2022). Ecological region division of soil based supplementary fertilization and decrement fertilization in China citrus orchards. J. Huazhong Agric. Univ. 41 (1), 9–19. doi: 10.13300/j.cnki.hnlkxb.2022.01.002

[B179] Xia Y. Quyang G. Sequeira R. A. Takeuchi X. Baez J. Chen J. (2011). A review of huanglongbing (citrus greening) management in citrus using nutritional approaches in China. Plant Health Prog. 12 (1), PHP-2010-1003-01-RV. doi: 10.1094/PHP-2010-1003-01-RV

[B180] Xie J. Xiong H. Niu R. Wang Y. Wang Y. Lali M. N. (2025). Nutrient acquisition efficient rootstocks improve zinc nutrition of top grafted citrus trees on calcareous soil. Front. Plant Sci. 16, 1615405. doi: 10.3389/fpls.2025.1615405 40822723 PMC12350133

[B181] Xu E. Liu T. Gu D. Zan X. Li J. Zhou K. (2024). Molecular mechanisms of plant responses to copper: from deficiency to excess. Int. J. Mol. Sci. 25 (13), 6993. doi: 10.3390/ijms25136993. PMID: 39000099 PMC11240974

[B182] Xu Z. Zhou Y. Liu R. Cui H. Tan J. Zhou W. (2025). Available medium and micronutrients in the soils of major citrus-producing areas in Southeast China. J. Environ. Manage. 389, 126078. doi: 10.1016/j.jenvman.2025.126078. PMID: 40483862

[B183] Yang Y. Qi C. Gu Y. Fang G. (2024). Use efficiency, reduction potential, and effects of fertilizers on carbon emissions in China's major citrus regions. Agriculture 14 (11), 1971. doi: 10.3390/agriculture14111971

[B184] Yang Y. Shi Y. Tong C. Zhang D. (2025). Effects and mechanism of auxin and its inhibitors on root growth and mineral nutrient absorption in citrus (trifoliate orange, Poncirus trifoliata) seedlings via its synthesis and transport pathways. Agronomy 15 (3), 719. doi: 10.3390/agronomy15030719

[B185] Yang W. Yang H. Ling L. Chun C. Peng L. (2023). Tolerance and physiological responses of citrus rootstock cultivars to boron toxicity. Horticulturae 9 (1), 44. doi: 10.3390/horticulturae9010044

[B186] Yu X. Feng L. Huang Y. Liang Y. Pan F. Zhang W. (2024). Planted citrus regulates the community and networks of phoD-harboring bacteria to drive phosphorus availability between Karst and non-Karst soils. Microorganisms 12 (12), 2582. doi: 10.3390/microorganisms12122582. PMID: 39770784 PMC11678004

[B187] Zang Y. Huang Y. Chang X. Chen J. Jiang T. Wu Z. (2023). High soil pH and plastic-shed lead to iron deficiency and chlorosis of citrus in coastal saline-alkali lands: a field study in Xiangshan county. Horticulturae 9 (4), 437. doi: 10.3390/horticulturae9040437

[B188] Zeng Q. Mei T. Wang M. Tan W. (2024). Linking phosphorus fertility to soil microbial diversity and network complexity in citrus orchards: implications for sustainable agriculture. Appl. Soil Ecol. 200, 105441. doi: 10.1016/j.apsoil.2024.105441

[B189] Zhang J. Huang W. Chen W. Rao R. Lai N. Huang Z. (2024). Mechanisms by which increased pH ameliorates copper excess in Citrus sinensis roots: insight from a combined analysis of physiology, transcriptome, and metabolome. Plants 13 (21), 3054. doi: 10.3390/plants13213054. PMID: 39519972 PMC11548300

[B190] Zhou G. Li B. Chen J. Yao F. Guan G. Liu G. (2020). Physiological and nutritional responses of 'HB' pummelo [Citrus grandis (L.) Osbeck 'Hirado Buntan'] to the combined effects of low pH levels and boron deficiency. HortScience 55 (4), 449–456. doi: 10.21273/HORTSCI14708-19

[B191] Zou Y. N. Wan Y. X. Zheng F. L. Cheng X. F. Hashem A. Wu Q.-S. (2025). Mycorrhizal trifoliate orange plants tolerate soil drought by enhancing photosynthetic physiological activities and reducing active GA_3_ levels. Tree Physiol. 45 (12), tpaf073. doi: 10.1093/treephys/tpaf073. PMID: 40590498

[B192] Zou Y. N. Wan Y. X. Zheng F. L. Cheng X. F. Hashem A. Wu Q.-S. (2025). Mycorrhizal trifoliate orange plants tolerate soil drought by enhancing photosynthetic physiological activities and reducing active GA_3_ levels. Tree Physiol. 45 (12), tpaf073. doi: 10.1093/treephys/tpaf073. PMID: 40590498

